# Sex differences in autoimmune diseases

**DOI:** 10.1186/2042-6410-2-1

**Published:** 2011-01-04

**Authors:** Rhonda Voskuhl

**Affiliations:** 1 Professor, UCLA Dept. of Neurology, Jack H Skirball Chair for Multiple Sclerosis Research, Director, UCLA Multiple Sclerosis Program, Neuroscience Research Building 1, Room 475D, 635 Charles Young Drive South, Los Angeles, CA 90095, USA

## Abstract

Women are more susceptible to a variety of autoimmune diseases including systemic lupus erythematosus (SLE), multiple sclerosis (MS), primary biliary cirrhosis, rheumatoid arthritis and Hashimoto's thyroiditis. This increased susceptibility in females compared to males is also present in animal models of autoimmune diseases such as spontaneous SLE in (NZBxNZW)F1 and NZM.2328 mice, experimental autoimmune encephalomyelitis (EAE) in SJL mice, thyroiditis, Sjogren's syndrome in MRL/Mp-lpr/lpr mice and diabetes in non-obese diabetic mice. Indeed, being female confers a greater risk of developing these diseases than any single genetic or environmental risk factor discovered to date. Understanding how the state of being female so profoundly affects autoimmune disease susceptibility would accomplish two major goals. First, it would lead to an insight into the major pathways of disease pathogenesis and, secondly, it would likely lead to novel treatments which would disrupt such pathways.

## Why study sex differences?

Translational research which starts with a clinical observation, such as the increased susceptibility to autoimmune disease in females, can be termed as a 'bedside to bench to bedside approach'. In contrast to the classic 'bench to bedside' approach, research that begins with a clinical observation carries less inherent risk of failure. In the 'bench to bedside' approach, a molecule of interest is focused upon as a target to either block or enhance because it is thought to be either disease promoting or inhibiting, respectively. This is an immune molecule in the case of autoimmune diseases. The inherent risk of this approach is that, while a molecule of interest may be key to a pathway in an *in vitro *culture, it may not be key *in vivo *in the animal model due to redundant molecular pathways that exist *in vivo*. Further, even when a molecule of interest appears to be important in disease pathogenesis in a given animal model, it may not be critical in human disease due to differences in disease pathways in the outbred human population. Thus, the vast majority of research avenues focusing on a given molecule of interest do not ultimately prove to be physiologically significant in human disease. This results in very high costs for research and development as only a few make it to the market as new treatments. In contrast, the 'beside to bench to bedside' approach begins with a clinical observation that is known to be physiologically relevant in the human disease. For example, in autoimmune disease, a major disease susceptibility factor is the state of being female. If this were understood, one would have discovered something that is indeed physiologically significant. In order to understand why being female confers increased susceptibility, one must next go to the bench, using *in vitro *and *in vivo *systems to simulate this clinical observation and dissect out its underlying aetiology. After one or more molecules have been discovered which are responsible for conferring increased susceptibility of females, one can then go back to the bedside in the form of a clinical trial to test whether targeting these molecules results in disease modification. Since the 'bedside to bench to bedside' research avenue initially began with a known clinical observation, the risk that treatments targeting pathways underlying this observation will be clinically insignificant is reduced.

Notably, the study of sex differences is important for both women and men with autoimmune diseases. The goal of this research is not merely to discern what is makes women more at risk but also what makes most men less at risk. If a factor were identified in men that confers disease protection in most, this may lead to insight into why some males do, indeed, develop autoimmune disease.

## Barriers to studying sex differences in translational research

Powerful tools have been developed to unravel mechanisms underlying sex differences in diseases. Sex hormones and/or sex-linked gene inheritance may be responsible for the enhanced susceptibility of females to autoimmune diseases. As will be discussed in detail below, genetically modified mice and molecular reagents can be used to separate out sex hormone effects from sex chromosome effects, as well as to identify which hormone receptor or sex chromosome gene is involved in a given process. Meanwhile, powerful observational analyses of sex differences in a range of diseases are beginning applied to data collected anecdotally as well as in clinical trials.

Why, in light of the wealth of data on clinical observations and the sophistication of tools needed to ascertain mechanisms of sex differences, are there so few current clinical trials based on sex differences research? A major obstacle is that large clinical trials are very costly and companies must be able to recover these costs by marketing products for the years during which patent protection exists. Indeed, patent products are in place for novel molecules of interest in the classic 'bench to bedside' approach extremely early during development. Unfortunately, treatments evolving from insights made through the study of sex differences may ultimately entail the use of a product which cannot be patented. For example, if a naturally occurring hormone is the ultimate treatment identified, it cannot be patent protected. Thus, even if trials with a hormone were successful clinically, costs for large clinical trials would be difficult to recover. Therefore, for financial reasons, once it becomes clear that a product patent is not attainable, development of a given product by a company can be either halted or, perhaps, not even pursued.

Development of products that cannot be patent protected would ultimately produce relatively inexpensive treatments. This would be good for healthcare costs at for both the individual and the population as a whole. While some sex differences basic research and clinical trials are funded by nonprofits agencies and philanthropy, the level is far less than that undertaken by the pharmaceutical industry.

Finally, finding a new use for an old drug, development of generics or other non-patentable products for a new indication has a safety advantage. These drugs have been used in hundreds of thousands of patients for decades and their safety profile is well characterized, often with vast post marketing experience. Novel molecules that are discovered, patented and developed by a pharmaceutical company are in their relative infancy with respect to their safety profile. Generally, they are initially given to a few subjects for a few weeks and then ultimately given to, at most, a few thousand subjects for 1-2 years in order to attain the Food and Drug Administration approval. Unfortunately, during the post marketing experience, after thousands of patients have been treated for longer periods of time, serous adverse effects and even some deaths can occur.

Overall, sex differences research can lead to new insights and treatments that will be more likely to have clinically significant effects, will be safer and less expensive.

## The approach for studying sex differences using multiple sclerosis (MS) as an example

This review will focus on the disease MS as an example of how one may use the 'bedside to bench to bedside' approach. It outlines how one may take advantage of the currently available information on clinical observations of sex differences in the disease, utilize powerful tools to dissect out basic mechanisms and then translate these findings to clinical trials. Thus, a brief description of MS and its disease model will be presented below.

MS is a putative autoimmune demyelinating disease. The target organ of the immune response is the central nervous system (CNS). Clinically, patients present with discrete episodes of neurological dysfunction which initially improve over weeks to months. This is a relapse. Hence, the early phase of the disease is the relapsing remitting phase (RR) of MS. The RR MS is characterized by large numbers of enhancing lesions on brain magnetic resonance imaging (MRI) which are thought to be biomarkers for inflammatory foci pathologically and relapses clinically. Enhancing lesions resolve after about 1-2 months and leave behind a hyperintensity on T2 weighted imaging that may reflect scars or a past lesion load. Clinically, over the course of weeks to months, complete or partial recovery of function occurs after a relapse. This RR phase of the disease is thought to be principally driven by the inflammatory lesions and, indeed, this early phase of MS has been shown to be responsive to anti-inflammatory treatments such as interferons [1-5]. Unfortunately, after years of carrying the diagnosis of RR MS (5-15 years), most patients 'transition' to a more permanently debilitating form of the disease. This later stage is called the secondary progressive phase (SP) of MS. Patients with SP MS have fewer enhancing lesions and fewer relapses, but the relapses they have are associated with poorer recovery. On magnetic resonance imaging MRI atrophy accumulation becomes apparent. There is also a progression of disability, even in the absence of clear relapses, and less responsiveness to anti-inflammatory medications [6,7]. There is a poor correlation with disability and inflammation on MRI and a better correlation between disability and atrophy. Anti-inflammatory therapies, which reduce enhancing lesions on MRI and clinical relapses, may delay atrophy and permanent disability accumulation if taken early in disease but they do not prevent the development of either. In summary, the RR MS phase is considered to be a more inflammatory phase of the disease, while the SP MS phase is a more neurodegenerative phase.

As is the case with most autoimmune diseases, one must consider the processes in both the immune response and the target organ. With regard to the immune response, MS has long been considered to be mediated by myelin protein-specific CD4+ T lymphocytes secreting T helper 1 (Th1) type cytokines such as interferon gamma (IFNγ) [8]. T lymphocytes and macrophages infiltrate the CNS and secrete pro-inflammatory cytokines such as IFNγ, interleukin-12 (IL-12), tumour necrosis factor (TNFα) and IL-17 which then sets off a cascade of events ultimately leading to demyelination of axons. This acute demyelination leads to a conduction block of neurons and clinical relapse results [9]. In contrast to the deleterious Th1 responses described above, Th2 responses, which include the production of cytokines such as IL-4, IL-5 and IL-10, are thought to be beneficial in MS. In murine systems, Th1 and Th2 immune responses are counter regulatory and, in states of health, the two responses exist in a delicate balance [10]. While there are clearly some differences in human and murine systems, therapies for MS have nevertheless aimed to either reduce Th1 or Th17 responses or to increase Th2 responses. While the currently available therapies with proven benefit in RR MS (interferon beta 1b, interferon beta 1a and copolymer-1) have numerous possible mechanisms of action, several reports indicate that they act, at least in part, through inducing favourable changes in the production of these cytokines [11-14]. Other mediators of MS include chemokines and adhesion molecules which play a role in the recruitment of immune cells from the blood to the CNS lesion foci. Tysabri, a drug which blocks adhesion molecules, was shown to reduce relapses in MS [15]. Other immune mechanisms in MS involve a variety of costimulatory molecules. Trials to determine if blocking co-stimulatory molecules may result in clinical benefit are ongoing. Finally, as autoantigen presentation by macrophages and dendritic cells in the peripheral immune system, as well as by CNS resident microglia or astrocytes, may be involved in ongoing inflammation, strategies to prevent antigen presentation by these cells are also being pursued as potential treatments.

As stated above, anti-inflammatory treatments have been shown to reduce relapses in MS but have had only a modest effect on halting the accumulation of permanent disability. Particularly later in the disease, disability accumulation has continued, despite robust and effective immunosuppression with chemotherapeutic agents [16]. Thus, MS has both an inflammatory and a neurodegenerative component in its pathogenesis. Over the last decade abundant neuroimaging and neuropathological studies have described the significant neurodegenerative process in MS. Neuroimaging has demonstrated atrophy [17-22], particularly in grey matter [21,23,24]. This grey matter atrophy has been shown to correlate better with permanent disability than the white matter inflammatory marker of gadolinium enhancing lesions [6,19,21]. Also, abnormalities beyond classic white matter T2 hyperintensities, within 'normal appearing white matter' (NAWM), have been seen using magnetization transfer, spectroscopy and diffusion weighted imaging [25-31]. Furthermore, the degree of change in the NAWM may be a predictor of future clinical progression [32]. Pathological findings in MS have described cortical lesions that were characterized by transected neurites (both axons and dendrites) and apoptosis. There was very little T and B cell infiltration. Activated microglia ensheathed apical dendrites, neurites and neuronal perikarya [33,34]. Axonal transection has also been described within white matter lesions raising the possibility of Wallerian degeneration in white matter tracts. In light of these clinical, neuroimaging and neuropathologic observations there is now a consensus by MS investigators that there is a need to discover a treatment which is primarily designed as a neuroprotective agent for MS. This neuroprotective agent should be taken in combination with an anti-inflammatory treatment. Theoretically, combination treatment with both an anti-inflammatory agent and a neuroprotective agent would have a greater impact upon the halting of permanent disability accumulation in MS compared to treatment with an anti-inflammatory agent alone.

### Sex differences in MS

During reproductive ages (18-40), there is a distinct female preponderance of autoimmune diseases including MS [35]. Sex hormones and/or sex chromosomes may be responsible for this enhanced susceptibility. In males, the onset of MS tends to be relatively later in life (30-40), coinciding with the beginning of the decline in bioavailable testosterone (T) [36]. Thus, the female to male ratio is greater in patients presenting with MS before 20 years old (3.2:1) compared to the ratio in the MS population as a whole (2:1) [37]. Interestingly, a recent Canadian study found a disproportionate increase in the incidence of MS in women [38]. This increase in the sex ratio, approaching 4:1, was theorized to possibly be of environmental origins. Alternatively, these data showing an increase in diagnosis of women with MS may merely be reflective of the propensity for neurologists to diagnose women with autoimmune disease. As time passes, neurologists have more sensitive tools for detecting early MS and with these come a growing appreciation of the importance of early treatment in slowing disability accumulation. Notably, the relatively higher sex ratios in MS recently reported remain consistent with the sex ratios that were previously known in other autoimmune diseases such as rheumatoid arthritis (RA) and systemic lupus erythematosus (SLE).

In general, it has been well documented that women have more robust immune responses than men [39]. Females have a greater humoral response, as evidenced by higher serum immunoglobulin concentrations than males and a greater antibody response to various antigens after immunization [40]. In addition, females reject skin allografts faster and have a reduced incidence of tumours, indicating that they also have a greater cellular immune response [35]. This is also true when comparing women and men with MS. Autoantigen (proteolipid protein) specific immune responses from women were characterized by higher levels of Th1 responses [41-43]. On the other hand, it is still a controversial whether the disease course differs between the sexes remains controversial. Despite the more robust immune responses in women, some studies have suggested that men may have a more progressive course of disease [36,44,45]. Further, early neuroimaging studies suggested that women have more inflammatory markers in the CNS, while men have more neurodegenerative outcomes [46]. However, when both the clinical and neuroimaging data were adjusted for age, these sex differences in progression and neurodegenerative outcomes became less apparent. Together, these observations suggest that, while susceptibility and immune responses are higher in women than in men with MS, the rate of progression and neurodegeneration may, surprisingly, be no different. Thus, sex differences in the CNS should be considered independently of sex differences in the immune system in MS, with the potential for opposing influences.

### The murine model of MS

While there is no perfect animal model of MS, experimental autoimmune encephalomyelitis (EAE) is the most widely used animal model for studying the pathogenesis of MS. Decades of work have described the immunology and the neuropathology of EAE. Immunologic studies focused on myelin protein specific immune responses including those directed against myelin basic protein (MBP), proteolipid protein (PLP) and myelin oligodendrocyte protein (MOG). Much of what has been theorized in MS with respect to immune responses was based on the initial findings in EAE, including the role for Th1 cytokines, IL17, chemokines, adhesion molecules and co-stimulatory molecules. Regarding the neuropathology of EAE, past studies focused on detailed characterization of lesions with respect to the inflammatory infiltrate composition, the level of demyelination, the level of oligodendrocyte loss, microglial activation and astrocytic scar formation [47-50]. Less attention has focused on neurons, with only recent descriptions of a relationship between axonal loss and disability [51]. Our group and others have each described CNS pathology 'beyond the lesion' in normal appearing white matter characterized by decreases in axon densities in white matter tracts of the dorsal spinal cord [52-54]. Another study described motor neuron dendritic abnormalites in grey matter of spinal cords of mice with EAE [55] and our group has published that neuronal staining in spinal cord grey matter is abnormal even early during the course of EAE [53]. Regarding neuroimaging 'beyond the lesion' in EAE, diffusion tensor imaging (DTI) changes have been described in dorsal cord and optic nerve by others [52,56] and cerebellar grey matter atrophy has been described by our group [57]. Thus, while EAE has been the prototypic Th1 mediated disease tool used by immunologists for decades, more recently, EAE has also been characterized with respect to its neurodegenerative component by neuroscientists.

### Sex differences in the murine model of MS

EAE in the SJL strain of mice has been characterized to have a sex bias that parallels that of multiple sclerosis, with males being less susceptible to the disease than females [58-62]. In part, this sex difference may result from the protective effects of testosterone in male mice, as suggested by studies demonstrating that the removal of physiologic levels of testosterone from male mice via castration increased disease susceptibility [63,64]. Also, in animal models of other autoimmune diseases, where a similar gender dimorphism exists [non-obese diabetic (NOD) mice, thyroiditis and adjuvant arthritis), castration was shown to increase the disease prevalence and/or the disease severity [65-68]. Further, testosterone levels have been shown to be decreased in male mice during EAE relapse [69]. Together, these data support the hypotheses that endogenous androgens may be protective at physiologic levels.

Notably, however, while the SJL, the ASW and the NZW strains of mice have an increased susceptibility to EAE in females compared to males, the B10.PL and PL/J have more susceptibility to disease in males compared to females [70], while there is no sex bias in the C57BL/6 strain [71,72]. Effects of removal of androgens in EAE have previously been shown to be dependent upon genetic background [72] and some autosomal gene linkages to susceptibility to MS have been identified in one gender but not the other [73]. Thus, in the outbred human population, the genetic background of some, but not all, individuals may be permissive to sex effects. Importantly, since overall there is a gender difference in many autoimmune diseases in humans, we hypothesize that the incidence of relatively permissive genetic backgrounds are likely to be prevalent, not rare, occurrences.

## Testicular hormones in the MS model

Sex differences in MS and the EAE model may be related to sex hormones, sex chromosomes or both: female sex hormones or male sex hormones may play a role. This section will discuss a possible role for male sex hormones.

To begin to test the role of endogenous androgens in MS, the EAE model was used. Since EAE in the SJL strain of mice had previously been characterized to have a gender difference that paralleled that of MS, with males being less susceptible to disease than females [61,62], the SJL strain was used. Protective effects of testosterone in male mice were shown by studies demonstrating that the removal of physiologic levels of testosterone from male mice via castration increased disease susceptibility [63,72]. Finally, testosterone levels were shown to be decreased in male mice during EAE relapse [69]. Together, these data support the hypotheses that endogenous androgens are protective at physiologic levels in these autoimmune disease models.

As the MS population is genetically heterogenous, it was then necessary to determine whether the protective effect of endogenous androgens was a phenomenon that was unique to the SJL strain. The C57BL/6 strain was used since there was no sex bias in EAE in this strain [70-72]. Disease severity did not differ between castrated and sham C57BL/6 male mice, thereby indicating that endogenous androgens are not protective in all genetic backgrounds. A lack of a protective effect of endogenous androgens in C57BL/6 mice was found to not be due to lower endogenous levels of androgens in the C57BL/6 strain as compared to the SJL strain. Thus, equivalent levels of endogenous androgens were shown to be protective in males of one genetic background but not another [72]. This is consistent with findings that the protection from disease observed in young male mice was affected by the strain of origin of the Y chromosome [74]. Autosomal loci have been identified that control susceptibility in several clinical subtypes of EAE in both males and females [75]. A locus on chromosome 13 (*eae 13*) was linked to susceptibility of monophasic remitting/non-relapsing EAE in males but not females. It was hypothesized that a sex specific effect at *eae 13 *might be responsive to testosterone levels and, thereby, give males a greater ability to control disease once it is initiated. Interestingly, it has been shown that one gene locus, *rsl*, in the region of *eae 13*, is capable of regulating sexually dimorphic traits [76]. Therefore it is possible that endogenous androgens may be protective in a given strain depending upon *eae 13 *allele inheritance. Some genetic backgrounds may also carry other alleles that are more sensitive to the effect of testosterone at a given endogenous level.

Notably, a lack of protection by endogenous androgens in a given genetic background did not preclude a therapeutic effect of supplemental androgen treatment in EAE. Thus, treatment was implemented using either testosterone or 5α-dihydrotestosterone (DHT). DHT was used as it cannot be converted to oestrogens. Indeed, not only were both androgen treatments shown to be protective in gonadally intact males of a strain in which endogenous androgens were protective (SJL) but they were also protective in a strain in which endogenous androgens were not protective (C57BL/6) [72]. These data indicated that, even in genetic backgrounds that are not permissive to a protective effect of endogenous androgens, protection can still be provided by supplemental exogenous androgen treatment. This suggested that the mechanisms of disease protection may differ between endogenous physiologic androgens and supplemental supraphysiologic androgen treatment. These preclinical data laid the groundwork for clinical trials that aimed to treat males in the heterogeneous MS population with testosterone.

Mechanisms underlying the protective effects of androgens have been previously investigated, and a number of studies have delineated immunological changes in androgen treated mice [77-80]. The balance between cytokines produced by Th1 and Th2 lymphocytes is considered central to the development of EAE with Th1 cytokines (IFN-γ and IL-12) being pro-inflammatory and Th2 cytokines (IL-10, IL-4 and IL-5) being anti-inflammatory [35]. *In vitro *treatment of naïve T cells from Vβ8.2 TCR transgenic mice stimulated with MBP Ac 1-11 peptide in the presence of testosterone resulted in increased production of IL-10 and IL-5 and decreased production of IFN-γ [79]. T cell lines from PLP 139-151 immunized mice stimulated with PLP 139-151 in the presence of DHT also resulted in increased IL-10 and decreased IFN-γ production [79]. *In vivo*, androgen treatment has also been shown to effect cytokine profiles. When splenocytes from mice with EAE treated *in vivo *with DHT or placebo were stimulated with MBP, those from DHT treated had increased IL-10 and decreased IFN-γ [77]. Further, when splenoctyes from healthy mice treated with DHT *in vivo *were stimulated with anti-CD3 antibody, increased levels of IL-10 and decreased levels IL-12 were observed [78]. Cytokine gene knockout mice were then used to show that DHT directly increased IL-10 from CD4^+ ^T lymphoctyes with a subsequent indirect decrease in IL-12 [78]. Together, these data indicated that one mechanism through which exogenous androgen treatment is protective in EAE involves effects on cytokine production.

## Testicular hormones in MS

Disease onset in females typically occurs soon after puberty. In males, disease onset usually occurs later in life (age 30-40), coinciding with the age at which serum testosterone levels begin to decline in normal healthy men [81-85]. Interestingly, this phenomenon may also be true of other autoimmune diseases. The onset of rheumatoid arthritis (RA) in men also takes place later in life with a reported fourfold increase in incidence rates in older men (ages 35-74) compared to younger men (ages 18-34). Further, the sex difference of RA in women versus men is 4:1 between ages 35-44, which decreases to 1.1:1, by age 75 [86]. Together, these observations suggested that relatively high levels of testosterone in young men after puberty may provide temporary protection from autoimmune disease onset in those men who were genetically predisposed to ultimately develop the disease.

Additionally, it has been reported that 24% of male MS patients tested had significantly lower levels of testosterone compared to age-matched healthy men [87]. The reason for the decreased testosterone levels remains unclear. It may be due to gonadal failure or to effects on the hypothalamic-pituitary axis (HPA) such as stress or hypothalamic lesions [69]. In men with MS and sexual dysfunction, low testosterone levels have been previously shown to be associated with low luteinizing hormone levels, thereby ruling out gonadal failure [69]. Interestingly, a decrease in free testosterone levels has been reported in untreated men with new onset RA [88], making the possibility of hypothalamic lesions in the brain in MS less likely. An effect of the stress of chronic illness on the HPA may be the most likely explanation for the relatively lower levels of testosterone in men with chronic autoimmune diseases.

Data in EAE suggested that supplemental, exogenous testosterone treatment may provide protection across genetic backgrounds in men of the heterogeneous MS population [72]. Thus, to investigate the possible effects of testosterone treatment in MS, testosterone was administered via transdermal application (AndroGel^®^) to men with RR MS in a small pilot trial [89]. A crossover design, using a within arm comparison of 6 months pre-treatment to 12 months treatment, was employed to reduce the effect of disease heterogeneity given the small sample size, as previously described in MS [90].

Ten subjects with RR MS completed the study. At baseline, all subjects had testosterone levels in the lower range of normal. During daily treatment with testosterone, serum testosterone levels rose 50% on average to the higher range of normal. Scores from the Paced Auditory Serial Addition Task (PASAT) component of the Multiple Sclerosis Functional Composite (MSFC), a commonly used cognitive test in MS, remained stable during the first 6 months of treatment (months 3 and 6), trended upward after 9 months and were significantly improved by month 12 of treatment. There was also a trend for improvement in spatial memory, consistent with previous reports of testosterone mediated improvements in working and spatial memory in healthy non-hypogonadal elderly men [91,92].

The 10 subjects had low levels of enhancing lesion activity on brain MRI at baseline and this low level was not significantly increased or decreased with treatment. The most interesting finding of the study was the effect on brain atrophy. There was a 67% reduction in the rate of brain volume loss during treatment compared to that seen at pre-treatment. Interestingly, the timing of the cognitive improvements coincided with the slowing of brain atrophy on MRI.

Finally, testosterone treatment was also shown to favourably affect the immune system by inducing decreasing CD4+ T cell percentage and increasing natural killer (NK) cells. In addition, peripheral blood lymphocytes (PBMC) production of IL-2 was significantly decreased while transformin growth factor (TGF)β1 production was increased. Furthermore, PBMCs obtained during the treatment period produced significantly more of neuroprotective factors brain-derived neurotrophic factor (BDNF) and platelet-derived growth factor (PDGF-BB) [93].

In summary, endogenous androgens are protective in EAE in some strains but not others. However exogenous supplemental treatment with androgens is protective across the genetic backgrounds. In humans with MS, we hypothesize that some, but not all, men may be of a genetic background that provides the protective effect of relatively high physiologic levels of testosterone that exist in young men. This may mask the early presentation of the relapsing remitting phase of the disease. As these men age, testosterone levels gradually decline and clinical MS onset is observed. Later, during the course of disease, it is possible that the stress of chronic disease may affect the hypothalamic pituitary axis which suppresses testosterone levels further, to within the low normal range. Further clinical trials are warranted in order to determine whether treatment with supplemental testosterone may offer some protection in men with MS.

## A second clinical observation

Ovarian hormones primarily include oestrogens and progesterone. Sex differences in MS could be due, at least in part, to sex differences in ovarian hormones. Indeed, the onset of MS in women is after puberty. However, there is no evidence that ovariectomy precipitates or aggravates MS. In contrast to the lack of evidence that endogenous cycling levels of ovarian hormones are major disease modifiers in MS, there is abundant evidence that the state of being pregnant is associated with disease protection. The effect of pregnancy appears to be due, in part, to a protective effect of high levels of oestrogen during late pregnancy. Thus, a second major clinical observation merges: pregnancy is a major disease modifier in MS. The study of sex differences in autoimmune disease includes: (1) research addressing female:male differences in a given autoimmune disease; and (2) research focused on a major disease modifier which is inherent to being one sex. Thus, in autoimmune disease, there are two major clinical observations. First, females are more susceptible than males. Second, pregnancy reduces relapses in females. Both of these observations can be pursued with respect to basic mechanism and translational potential.

With regard to this second clinical observation, it has been appreciated for decades that symptoms of patients with autoimmune diseases are affected by pregnancy and the post partum period. The most well characterized observations include those seen in MS, RA and psoriasis. These patients experience clinical improvement during pregnancy with a temporary 'rebound' exacerbation post partum [35,94-100]. Interestingly, women with SLE may experience an exacerbation of symptoms with gestation [101]. This differential effect of pregnancy on these diseases is thought to reflect the difference in immunopathogenesis of SLE as compared to MS and RA.

What is the precise effect of pregnancy on MS? What had been generally recognized for decades was that there was a period of relative 'safety' with regard to relapses during late pregnancy followed by a period of increased relapses post partum. These clinical observations were supported by a small study of two patients who underwent serial cerebral MRIs during pregnancy and post partum. In both women there was a decrease in MR disease activity (T2 lesion number) during the second half of pregnancy and a return of MR disease activity to pre-pregnancy levels in the first months postpartum [102]. Other studies found that, in addition to having a decrease in disease activity in patients with established MS, the risk of developing the first episode of MS was decreased during pregnancy compared to non-pregnant states [100]. The most definitive study of the effect of pregnancy on MS came in 1998 by the Pregnancy in Multiple Sclerosis (PRIMS) Group [96]. Relapse rates were determined in 254 women with MS during 269 pregnancies and for up to 1 year after delivery. Relapse rates were significantly reduced from 0.7 per woman year in the year before pregnancy to 0.2 during the third trimester. Rates then increased to 1.2 during the first 3 months post partum before returning to pre-pregnancy rates. No significant changes were observed between relapse rates in the first and second trimester compared to the year prior to pregnancy. Together, these data clearly demonstrated that the latter part of pregnancy is associated with a significant reduction in relapses, while there is a rebound increase in relapses post partum.

Since mechanisms of action of the approved therapies for MS involve anti-inflammatory effects and, as these treatments result primarily in a reduction in relapse rates, it is logical to hypothesize that mechanisms underlying the protective effect of pregnancy on MS relapses involve anti-inflammatory effects. Pregnancy is a challenge for the immune system. From the mother's standpoint, the fetus is an allograft as it harbours antigens inherited from the father. It is evolutionarily advantageous for the mother to transiently suppress cytotoxic, cell mediated, Th1 type immune responses involved in fetal rejection during pregnancy. However, not all immune responses should be suppressed as humoral; Th2 type immunity is needed for the passive transfer of antibodies to the fetus. Thus, a shift in immune responses with a downregulation of Th1 and an upregulation of Th2 is thought to be necessary for fetal survival [10,103-105]. Indeed, it has been shown that, in both mice and humans, failure to shift immune responses in this manner results in an increase in spontaneous abortion [104,106,107]. This shift in immune responses from Th1 to Th2 occurs both locally at the maternal fetal interface [10,108,109] as well as systemically [104,106,107,110-112]. The systemic shift away from Th1 and toward Th2 was initially shown in murine systems by a decrease in mixed lymphocyte reactions of splenocytes and an increase in antibody production during pregnancy [112]. Antigen stimulated splenocytes were then shown to produce less Th1 cytokines and more Th2 cytokines when derived from pregnant mice [106,110]. In humans, peripheral blood mononuclear cells in women with successful pregnancies produced IL10 but no IFNγ, upon stimulation with trophoblast antigens [104]. In another study, antigen- and mitogen-stimulated PBMC derived from patients with normal pregnancies demonstrated a decrease in the production of IL2 and IFNγ and an increase in production of IL4 and IL10, with the lowest quantities of IL-2 and IFNγ and the highest quantities of IL4 and IL10 present in the third trimester of pregnancy [107]. During the third trimester pregnancy, *ex vivo *monocytic IL12 production was also found to be about threefold lower and TNFα production was approximately 40% lower than postpartum values [111]. Two recent studies have been completed where MS subjects were followed longitudinally for immune responses during pregnancy and post partum. Gilmore *et al*. demonstrated that *ex vivo *stimulated PBMCs had increased IFNγ production post partum as compared to the third trimester and myelin protein specific T cell lines derived from subjects in the third trimester produced more IL10 [113], while Al-Shammri *et al*. found that six of the eight MS patients' *ex vivo *stimulated PBMCs showed a distinct shift from a Th2 cytokine bias (IL4 and IL10) during pregnancy, towards a Th1 cytokine bias (IFNγ and TNFα) after delivery [114]. In light of these data which demonstrated a relative shift to Th2 systemically during pregnancy, with a rebound back to Th1 post partum, it becomes highly plausible that these alterations in the immune response could underlie the improvement in putative Th1 mediated autoimmune diseases during pregnancy, as well as the exacerbation post partum.

Pregnancy is a complex event characterized by changes in numerous factors which may impact the CNS. These include increases in oestriol, oestradiol, progesterone and prolactin, to name a few [115]. Potential neuroprotective effects of oestrogens and progesterone will be discussed in depth below. Recently, it was shown that pregnant mice have an enhanced ability to re-myelinate white matter lesions and that prolactin regulates oligodendrocyte precursor proliferation and mimics this regenerative effect of pregnancy [116]. This finding may be potentially exploited in pursuit of a therapeutic repair strategy to increase remyelination. However, when considering the effect of prolactin treatment in neuroimmunologic diseases such as MS, one must recall that prolactin has pro-inflammatory properties which can exacerbate disease, as has been shown in the MS model, EAE [117]. Thus, when considering any pregnancy factor, its effects on both the CNS and the immune system must be considered. Notably, the clinical observation that breast feeding, which would be associated with a prolonged state of increased levels of prolactin, had no effect compared to no breast feeding with respect to the post partum rate in MS [118]. A lack of a difference in relapse rate may suggest that the pro-inflammatory properties of increased prolactin during breast feeding may not be clinically significant. On the other hand, the relapse rate is a relatively insensitive, albeit important, outcome measure. Future studies should compare immune responses, enhancing lesions on MRI and prolactin levels in MS women who are, or are not, breast feeding post partum.

What is striking about pregnancy is that it represents a state that is characterized by two important changes. First, there is a downregulation of cellular immune responses. This relative immunosuppression probably occurs in order to prevent fetal rejection. Second, pregnancy is characterized by the presence of potentially neuroprotective hormones, such as oestrogens, progesterone and prolactin. It would seem evolutionarily advantageous to have such neuroprotective factors present as neuronal and oligodendrocyte lineage cells in the fetus progress through critical developmental windows [119]. Together, the combined anti-inflammatory and neuroprotective state of pregnancy, which is, perhaps, aimed at protecting the fetus, is precisely what is needed to protect the CNS during inflammatory attack in MS.

## Treatment with ovarian hormones in EAE

It was shown over a decade ago that EAE in guinea pigs, rats and rabbits improved during pregnancy [94]. Further, it was shown that relapsing-remitting EAE in SJL mice improved during late pregnancy [120,121]. The EAE model was then used to determine if an increase in levels of a certain hormone during pregnancy might be responsible for disease improvement. Since oestrogens and progesterone increase progressively during pregnancy to the highest levels in the third trimester, these hormones were candidates for possibly mediating a protective effect. Two oestrogens, oestradiol and oestriol, increase progressively during pregnancy. Oestradiol is otherwise present at much lower fluctuating levels during the menstrual cycle in non-pregnant women and female mice. Oestriol, in contrast, is made by the fetal placental unit and is not otherwise present in non-pregnant states. Over the last 10 years it has been shown in numerous studies that oestrogen treatment (both oestriol and oestradiol) can ameliorate both active and adoptive EAE in several strains of mice (SJL, C57BL/6, B10.PL, B10.RIII) [122-130]. Oestriol treatment has also been shown to be effective in reducing clinical signs in EAE when administered after disease onset [129]. Finally, both oestradiol and oestriol have been shown to be efficacious in both female and male mice with EAE [131].

Effects of oestrogen treatment in animal models of RA are similar to those in EAE. Oestrogen treatment is clearly protective in collagen induced arthritis (CIA) and pharmacologic blocking of both nuclear ERα and ERβ receptors abolished this protection [132-135]. In contrast, oophorectomy ameliorates murine lupus, while oestrogen treatment, even at physiologic doses, accelerates disease [136-138]. The mechanism for the deleterious effect of oestrogen on murine lupus appears to involve, at least in part, an oestrogen induced effect on genes involved in survival and apoptosis of B cells which results in a skewing of naïve B cells towards autoimmunity [139-142]. Opposite effects of oestrogen treatment in EAE and CIA, as compared to oestrogen treatment in lupus, are reminiscent of the opposite effects of pregnancy in the human autoimmune diseases they model.

The type and dose of the oestrogen used in a disease appears to be critical. A clinical amelioration of EAE occurred when oestriol was used at doses to induce serum levels which were physiologic with pregnancy. On the other hand, oestradiol had to be used at doses several fold higher than pregnancy levels in order to induce the same degree of disease protection [128]. Thus, while it is clear that high doses of oestradiol are protective in EAE, it has not yet been clearly established whether low doses of oestradiol are protective. Due to major differences in metabolic rates between humans and mice, it is very difficult to determine what a low dose oestrogen in an oral contraceptive pill in humans would equate to in a mouse. Thus, one can only use available physiologic benchmarks, such as the dose needed to induce a level in blood equal to that in pregnant mice or the dose needed to induce an oestrus level in an ovariectomized mouse. Rigorous comparisons of blood levels in pregnant or oestrus mice, assessed in parallel with levels in oestradiol treated mice are needed. Since ovariectomy removes physiologic levels of oestradiol, as well as progesterone, data on the effect of ovariectomy on EAE is somewhat informative in this regard. Some reports have found that ovariectomy of female mice makes EAE worse [125], while others have found that ovariectomy does not have a significant effect on disease [121]. Thus, it is controversial whether low levels of endogenous oestradiol which fluctuate during the menstrual cycle have a significant influence on EAE.

What is the effect of the other major ovarian hormone, progesterone, in EAE? Theoretically progesterone treatment could be beneficial as it had previously been shown in other systems to aid in myelination by oligodendrocytes [143,144]. However, while the severity of EAE was significantly reduced in oestriol or oestradiol treated mice as compared to placebo treated, treatment with progesterone was indistinguishable from the placebo [129]. A variety of progesterone doses, ranging from low (physiologic with menstrual cycle levels) to moderate (physiologic with late pregnancy levels) to very high (supraphysiologic), were used in combination with oestrogen (either oestradiol or oestriol) in EAE and none of the progesterone doses significantly altered the protective effect of oestrogen treatment on EAE disease course [121]. The lack of an effect of progesterone treatment on EAE was somewhat disappointing since progesterone had been shown to enhance remyelination *in vitro *[144-146]. Interestingly, in the Lewis rat, progesterone treatment alone was shown to deleterious, causing increased motor deficits, increased inflammation and increased neuronal apoptosis during acute EAE, while oestrogen treatment in combination protected against these deleterious effects of progesterone [147]. These data in EAE are consistent with previous work by the Holmdahl group in the mid 1990s which demonstrated that treatment with oestrogens could ameliorate collagen induced arthritis [128,132,135], while progesterone treatment alone had no effect. Progesterone treatment had only mild synergistic effects when used in combination with oestrogen treatment [148]. Together, these data indicate that oestrogen, not progesterone, can mediate disease protection during EAE.

Protective mechanisms of oestrogen treatment (both oestriol and oestradiol) in EAE clearly involve anti-inflammatory processes, with oestrogen treated mice having fewer inflammatory lesions in the CNS [129]. In adoptive EAE in SJL mice, an increase in IL10, with no change in IFNγ, was observed in *ex vivo *stimulated MBP specific responses, which was accompanied by an increase in MBP specific antibody of the IgG1 isotype, with no change in those of the IgG2a isotype [129]. In active EAE in C57BL/6 mice, a decrease in TNFα, IFNγ, and IL6 has been observed, with an increase in IL5. [122,123,130,131]. Oestrogen treatment has also been shown to downregulate chemokines in the CNS of mice with EAE and may affect expression of matrix matalloprotease-9 (MMP-9), each leading to impaired recruitment of cells to the CNS[125,127]. Oestradiol at pregnancy levels, but not below, reduced IL17 and increased PD-1 in T regulatory cells [149]. In addition oestrogen treatment has been shown to impair the ability of dendritic cells to present antigen [124,150]. The effect of oestrogen treatment on dendritic cell function in EAE may involve upregulated expression of indoleamine 2,3-dioxygenase (IDO) [151]. IDO is an enzyme involved in the catabolism of tryptophan which is expressed in antigen-presenting cells of lymphoid organs and in the placenta. It was shown that IDO prevents rejection of the fetus during pregnancy, probably by inhibiting alloreactive T cells [152] and it has been shown that IDO expression in antigen-presenting cells may also control autoreactive immune responses [153]. Finally, oestrogen treatment has recently been shown to induce CD4+CD25+ regulatory T cells in EAE [154,155]. Thus, oestrogen treatment has been shown to be anti-inflammatory through a variety of mechanisms.

An anti-inflammatory effect of oestrogen treatment in EAE does not preclude an additional more direct neuroprotective effect. Oestrogens are lipophilic, readily traversing the blood brain barrier, with the potential to be directly neuroprotective [156-158]. Numerous reviews have described oestrogen's neuroprotective effects, both *in vitro *and *in vivo *[157-159] in other model systems. *In vitro*, oestrogens have been shown to protect neurons in a variety of models of neurodegeneration, including those induced by excitotoxicity and oxidative stress [160-163]. Treatment with oestrogen decreased glutamate-induced apoptosis and preserved electrophysiologic function in neurons [159,164,165]. Decreased microglial activation has also been demonstrated after oestrogen treatment [166-168], along with modulation of the astrocytic response to injury [169,170]. Oestrogen treatment also protected oligodendrocytes from cytotoxicity [171-173] as well as accelerated oligodendrocyte process formation [174]. *In vivo *studies have shown that oestrogen treatment can be neuroprotective in animal models of Parkinson's disease, cerebellar ataxia, late onset leukodystrophy, stroke and spinal cord injury, often by reducing apoptosis via upregulation of the anti-apototic genes bcl-2 and bcl-x [158,159,175-181]. Oestrogens have also been shown *in vitro *and *in vivo *to increase dendritic spine formation and synapses on CA1 pyramidal cells of the hippocampus in healthy rats, resulting in improved working spatial memory [182-186].

Given the neuroprotective effect of oestrogen treatment in other disease models, it was then addressed whether oestrogen treatment might be neuroprotective in EAE, the MS model. Oestradiol treatment not only reduced clinical disease severity and was anti-inflammatory with respect to cytokine production in peripheral immune cells, it also decreased CNS white matter inflammation and demyelination. In addition, decreased neuronal staining (NeuN+/β3-tubulin+/Nissl), accompanied by increased immunolabelling of microglial/monocyte (Mac 3+) cells surrounding these abnormal neurons, was observed in grey matter of spinal cords of placebo treated EAE mice at the earliest stage of clinical disease, and treatment with oestradiol significantly reduced this grey matter pathology. Thus, oestradiol treatment was not only anti-inflammatory but also neuroprotective in the prevention of both white and grey matter pathology in spinal cords of mice with EAE [53].

Determining which oestrogen receptor mediates the protective effect of an oestrogen treatment in disease is of central importance for future development of selective oestrogen receptor modifiers which aim to maximize efficacy and minimize toxicity. The actions of oestrogen are mediated primarily by nuclear oestrogen receptors, ERα and ERβ, although non-genomic membrane effects have also been described [187]. Originally it was thought that ERα and ERβ would each have distinct tissue distributions, thereby providing a means through which use of selective oestrogen receptor modifiers (SERMs) could improve tissue selectivity. However, the relationship between ERα and ERβ became complex, with most tissues expressing detectable level of each of these receptors [188]. The two receptors at times did, and at other times did not, co-localize to the same cells within a given tissue [189,190]. Further, in some tissues the two receptors were shown to act synergistically, while in other tissues they acted antagonistically. Tissue specific differences in biologic outcomes are thought to be due, in part, to tissue specific differences in transcription factors which become activated upon binding of each oestrogen receptor (ER) by ligand [191-193].

Despite the fact that both ERα and ERβ are expressed in both the immune system and the CNS [188,189,194,195], oestrogen receptor knock out mice have been used to show that the protective effect of oestrogen treatment (oestradiol and oestriol) in EAE was dependent upon the presence of ERα, not ERβ [126,130]. However, whether targeted stimulation of ERα in developmentally normal mice was both necessary and sufficient for the oestrogen mediated protection in EAE remained unknown. A highly selective ERα ligand, propyl pyrazole triol (PPT) [196], was then used to determine whether stimulation of ERα in developmentally normal mice would be sufficient for the oestrogen mediated protection in EAE. Our group [53], and another [197], have each found that treatment with this ERα selective ligand was indeed sufficient for protection in EAE and was capable of inducing favourable changes in autoantigen specific cytokine production in the peripheral immune system (decreased TNFα, IFNγ and IL6, with increased IL5), as well as decreasing pathology in the CNS.

Subsequently, bone marrow chimeras were used by Garidou *et al*. to show that the disease ameliorating effects of oestradiol treatment in EAE were not dependent upon ERα signalling in blood derived inflammatory cells [198]. This suggested that the functionally significant target for oestrogen's actions *in vivo *during EAE is cells within the CNS. Given that treatment with either oestradiol or the ERα ligand was both anti-inflammatory and neuroprotective, the central question then became whether the neuroprotective properties of oestrogen treatment in EAE were or were not dependent upon the anti-inflammatory properties. Notably, a study had suggested that an anti-inflammatory effect was necessary to observe oestrogen mediated neuroprotection in stroke [199].

Since treatment with an ERα ligand is so anti-inflammatory in the peripheral immune system of EAE mice, there is a dramatic reduction in immune cells going into the CNS. While myelin and axons are preserved in the CNS, it cannot be discerned whether the effect of ERα ligand treatment is solely anti-inflammatory (and only indirectly neuroprotective) versus whether it is both anti-inflammatory and directly neuroprotective. In this context, direct neuroprotection refers to protection of CNS cells which is not merely due to reducing the immune attack. It is very difficult to treat only the CNS with an ERα ligand and block all effects in the periphery. However, differential neuroprotective and anti-inflammatory effects of ERα and ERβ ligand treatment revealed insights into whether oestrogen treatment might be directly neuroprotective in EAE [54]. Clinically, ERα ligand treatment abrogated disease at the onset and throughout the disease course. In contrast, ERβ ligand treatment had no effect at disease onset, but promoted recovery during the chronic phase of the disease. ERα ligand treatment was anti-inflammatory in the systemic immune system, while ERβ ligand treatment was not. However, treatment with either the ERα or the ERβ ligand was neuroprotective as evidenced by reduced demyelination and preservation of axon numbers in white matter [200,201]. Thus, by using the ERβ selective ligand, the anti-inflammatory effect from the neuroprotective effect of oestrogen treatment were dissociated and it was shown that neuroprotective effects of oestrogen treatment were not necessarily dependent upon anti-inflammatory properties.

Currently, it is unknown whether the neuroprotective effects of oestrogen treatments in EAE are directly mediated by binding oestrogen receptors on neurons or are indirectly mediated by binding oestrogen receptors on non-neuronal CNS cells. While neurons express oestrogen receptors and could be a target for oestrogen's actions, glial cells (oligodendrocytes, astrocytes and microglia) also express oestrogen receptors [202]. Myelin/axonal interactions, as well as astrocytic support for neurons, each make it possible that oligodendrocytes or astrocytes, respectively, may be the targets for oestrogen's protective effect on neurons and axons *in vivo*. Further, the neuroprotective effect of estrogens *in vivo *during EAE could also be mediated by reducing microglia activation since microglial activation can be deleterious for neurons.

## Treatment with ovarian hormones in MS

Levels of oestrogens that are lower than those which occur during pregnancy, such as levels induced by doses in oral contraceptives or hormone replacement therapy, may or may not be high enough to be protective in MS. While some studies have attempted to simulate a situation of treatment with oral contraceptives in EAE mice and have shown an effect on disease [122,127], doses used in mice are not readily translatable to humans. In fact, the data in humans thus far has suggested that treatment with oral contraceptives is not likely to suppress MS. The incidence rate of MS onset in both former and current oral contraceptive users was not different from that in never-users [203]. It is not surprising that former use of oral contraceptives in healthy women would have no effect on subsequent risk to develop MS, since one would not anticipate that the effect of treatment on the immune system would be permanent. However, the fact that incidence rates for MS in current oral contraceptive pill users were not decreased, compared to never-users, suggests that the oestrogens in oral contraceptives are not of sufficient type or dose to ameliorate the immunopathogenesis of MS, even temporarily, during current use. This remains an unresolved issue as controversial results emerge with respect to the use of oral contraceptives and MS risk during current use [204,205]. Studies of hormone replacement therapy and effects on disease activity in rheumatoid arthritis can provide further clues with respect to which doses of oestrogens could potentially be protective in MS [206]. In a randomized placebo controlled trial of transdermal oestradiol in 200 postmenopausal RA patients, who continued other anti-rheumatic medications, it was found that those who achieved a serum oestradiol level > 100 pmol/L had significant improvements in articular index, pain scores, morning stiffness and sedimentation rates, while those with lower oestradiol levels did not demonstrate improvement [207]. There has been no consistent correlation between disease markers in MS or RA and hormone levels during the menstrual cycle in females. Together, these reports suggest that it is probable that a sustained level of a sufficient dose of an oestrogen will be necessary to ameliorate disease activity in MS and RA.

Since oestriol is the major oestrogen of pregnancy and since an oestriol dose which yielded a pregnancy level in mice was protective in disease [129], oestriol was administered in a prospective pilot clinical trial to women with MS, in an attempt to recapitulate the protective effect of pregnancy on disease [208]. A crossover study was used whereby patients were followed for 6 months pre-treatment to establish baseline disease activity which included cerebral MRI every month and neurologic exam every 3 months. The patients were then treated with oral oestriol (8 mg/day) for 6 months and then observed for 6months or more months in the post treatment period. There were six RR patients and four SP patients who finished the 18 month study period. The RR MS subjects were then retreated with oral oestriol and progesterone in a four month extension phase. Oestriol treatment resulted in serum oestriol levels which approximated levels observed in untreated healthy control women who were 6 months pregnant. Interestingly, a significant decrease in a prototypic *in vivo *Th1 response, the delayed type hypersensitivity response to the recall antigen tetanus, was observed at the end of the 6 month treatment period as compared to the pre-treatment period. When PBMCs were stimulated *ex vivo*, a favourable shift in cytokine profile (decreased TNFα, increased IL10 and IL5) was observed during treatment as compared to baseline [209]. On serial MRIs, the RR patients demonstrated an 80% reduction in gadolinium enhancing lesions within 3 months of treatment, compared to pre-treatment [208] and this improvement in enhancing lesions correlated with the favourable shift in cytokine profiles [209]. Importantly, gadolinium enhancing disease activity gradually returned to baseline in the post treatment period and the favourable cytokine shift also returned to baseline. Further, in the 4 month extension phase of the study, both the decrease in brain enhancing lesions and the favourable immune shift, returned upon retreatment with oestriol in combination with progesterone in the RR MS group. These latter data have important translational implications since progesterone treatment is needed in combination with oestrogen treatment to prevent uterine endometrial hyperplasia when oestrogens are administered for a year or more in duration. These results indicate that treatment with progesterone in combination with oestriol did not neutralize the beneficial effect of oestriol treatment on these biomarkers of disease. A multi-centre, double-blind, placebo-controlled trial of oestriol treatment in RR MS is now ongoing in the USA.

Now we will review, and attempt to reconcile, findings of oestrogen treatment in basic science with oestrogen treatment in clinical trials in other diseases, mostly neurodegenerative diseases. Despite very promising results in various *in vitro *and *in vivo *animal systems describing neuroprotective effects of oestrogen treatment (reviewed above), a large study of oestrogen replacement therapy in mild to moderate Alzheimer's disease, with subjects an average age of 75 years, yielded disappointing results with no significant effects on clinical outcomes [210]. It has been hypothesized that either the age of the subject population, or the stage of disease, may be critical in whether a neuroprotective effect of oestrogen treatment can be appreciated [211]. Adequate levels of oestrogen receptors, or associated intracellular cofactors, may no longer be optimally expressed in either hormonally senescent, or significantly diseased, brains. Indeed the effect of oestrogen treatment on the blood brain barrier has been shown to be quite different when comparing senescent versus younger rats [212]. Clinical reports also indicate differential effects of oestrogen treatment depending on age and disease status. Oestrogen replacement had no effect on cognition in elderly menopausal women [213], while it preserved cognition in younger women undergoing surgical hysterectomy [214,215]. In a large trial assessing the effects of oestrogen treatment in stroke, treatment had no effect on cognition in women with stroke who had cognitive impairment at baseline but treatment reduced the risk for cognitive decline in those with normal cognition at baseline [216]. Finally, an improvement in cognitive function was observed with oestriol treatment in early RRMS, but not in late SPMS [208]. A 'healthy cell bias of oestrogen action' has been hypothesized to explain the disparity between promising results of oestrogen treatment in animal models of neurodegenerative diseases versus disappointing results in oestrogen treatment in some neurodegenerative disease trials [217]. Efficacy of oestrogen treatment appears to depend critically upon its administration early, as a preventative therapy, before neurodegeneration has occurred [210]. Also, results may be more promising if there is not a prolonged period of hypoestrogenicity before treatment with oestrogens [199]. Plaques in coronary arteries, a biomarker for cardiovascular risk, were reduced when conjugated equine oestrogens were administered relatively early to women 50-59 who had undergone hysterectomy [218]. The question is then prompted regarding whether oestrogen treatment might also need be to instituted relatively early in autoimmune or neurodegenerative diseases. Together, these data warrant further study of treatments with oestrogens, in MS and other diseases, which consider the age and disease duration of the subjects.

In summary, there has been shown that there is a protective effect of relatively high doses of oestrogens (oestradiol and oestriol) within the late pregnancy range in the MS model, while a role for lower endogenous fluctuating levels of estrogens during the menstrual cycle are controversial. In addition, there is no evidence that progesterone treatment is protective in the MS model. Mechanisms underlying the protective effects of high dose oestrogens include both anti-inflammatory and neuroprotective properties. Whether treatment with supplemental oestrogens may be protective in women with MS is currently being investigated through clinical trials.

## Sex chromosome effects in autoimmune disease

A non-mutually exclusive alternative to sex hormone-induced differences in autoimmune disease is a direct genetic effect on the immune system. That is, specific gene products, which are not induced by gonadal hormones but are expressed in a sexually dimorphic manner in the immune system, could induce sex-specific patterns of immune system development or function.

In male mammals, the Y-linked gene *Sry *is expressed in cells of the undifferentiated gonadal ridges to cause them to differentiate into Sertoli cells, which begins the differentiation of the testes [219]. Once the testes have formed, they secrete hormones distinct from those of the ovary and these hormonal differences generate sex differences in many non-gonadal tissues such as the external genitalia, immune system and central nervous system. Indeed, the effects of these hormones account for the majority of sex differences in non-gonadal tissues identified to date. However, there are other genetic differences between males and females arising from the difference in sex chromosome complement that could also contribute to sex differences in phenotype [220,221]. There are other Y genes whose role in non-gonadal tissues has been little studied and there are also differences in X gene expression arising from the difference in X chromosome complement. As it has been much easier to study the effects of gonadal hormones than these possible direct effects of X and Y genes, the sexually differentiating effects of hormones are much better understood. The focus of this section will be on whether direct actions of X or Y genes on tissues may also be important in autoimmunity.

As gonadectomy gives only limited information about putative effects of genes on sex chromosomes, we turned to a mouse strain that had been used by investigators studying sexually dimorphic development [221-226]. In these mice, the testis-determining gene *Sry *is 'moved' from the Y chromosome to an autosome by successive deletion from the Y chromosome and introduction of an *Sry *transgene onto an autosome. Thus, the inheritance of genes causing testicular differentiation is separated from the Y chromosome, and the Y chromosome can be present or absent in phenotypic male (testis-bearing) mice and it can be present or absent in phenotypic female (ovary-bearing) mice. The Y chromosome deficient in the *Sry *gene is denoted Y^- ^and XY^-^*Sry *male mice carry the *Sry *transgene. When this mouse is mated with an XX female, four progeny result: XX females, XY^- ^females, XX*Sry *males and XY^-^*Sry *males.

In order to determine the role of genes on sex chromosomes on autoantigen specific immune responses, we backcrossed the outbred MF1Y^-^*Sry *mice onto the SJL strain. We chose the SJL strain because a sex bias had been clearly shown in the SJL, with female SJL mice more susceptible than males to EAE [60-62,77,227]. Further, autoantigen specific immune responses had been shown to differ between females and males immunized with either MBP or PLP, with greater levels of Th1 cytokines produced in draining lymph node cells (LNCs) of females as compared to males [61,79,80,227,228]. In order to address direct effects of sex chromosomes on immune responses, the immune responses would be examined in SJL mice which differed in the complement of sex chromosomes, while having the same gonadal type. Gonadal females (XX and XY^-^) were ovariectomized prior to immunization to remove an effect of adult circulating female hormones on immune responses. Immune responses were significantly higher in ovariectomized XY^- ^mice compared to ovariectomized XX mice. These included increased levels of IFNγ, TNFα and IL-10 in supernatants of autoantigen stimulated immune cells. Further, when gonadal male mice were castrated prior to immunization, immune responses were significantly higher in castrated XY^-^*Sry *mice as compared to castrated XX*Sry *mice. While these data are consistent with an immunostimulatory role for a gene on the X chromosome [39], these findings cannot distinguish from an immunosuppressive effect of a gene on the Y chromosome [229].

The finding that the XY^- ^sex chromosome complement, compared to the XX, was relatively more stimulatory with respect to cytokine responses (IFNγ, TNFα, IL10) during autoantigen specific stimulation supported the concept of direct effects of sex chromosomes on autoimmunity, but the precise role in autoimmune disease remained unknown. IFNγ and TNFα may have either disease promoting or disease protective effects depending on the dose and timing of their production, while IL10 is generally considered to be protective in EAE. In addition, there are numerous other cytokines, chemokines, adhesion molecules and co-stimulatory factors that play a role in EAE pathogenesis and they, too, could theoretically be affected by sex chromosome complement. Thus, to directly assess the role of sex chromosome complement in disease, EAE was induced in mice with the informative sex chromosome complements. Specifically, both females and males were gonadectomized to remove any intercurrent effects of sex hormones which might mask effects of sex chromosomes [230]. SJL castrated male mice that were either XX*Sry *or XY^-^*Sry*, had active EAE induced with proteolipid protein peptide (PLP) 139-151 peptide. Clinical disease course was significantly more severe in XX*Sry *mice, as compared to XY^-^*Sry *mice. This significant difference in disease severity also occurred when comparing ovariectomized female XX versus XY^- ^mice. Further, in adoptive EAE, CNS pathology revealed higher levels of inflammation in mice that had received autoantigen specific cells derived from XX mice compared to those from XY^- ^mice. Together, these data showed that the XX sex chromosome complement, as compared to the XY^-^, was associated with a greater ability to induce EAE [231].

As it had been previously shown that no sex bias existed in EAE in the C57BL/6 strain of mice [70-72], it was then ascertained whether an effect of sex chromosomes could be found in this strain. Thus, the *Sry *deficient Y chromosome from the original outbred MF1 mice [232] was backcrossed onto the C57BL/6 strain. Both females and males were gonadectomized in order to remove any effects of sex hormones which might mask the effects of sex chromosomes. C57BL/6 castrated male mice that were either XX*Sry *or XY^-^*Sry *had active EAE induced with (MOG) 35-55 peptide. In contrast to results in the SJL, clinical disease courses were no different when comparing XX*Sry *mice with XY^-^*Sry *mice [231]. There was also no difference in disease when comparing ovariectomized female XX versus XY^- ^mice. Together, these data indicated that, when a strain was used that was characterized by a female to male difference in EAE (the SJL), a sex chromosome effect was present. In contrast, when a strain was used that is not characterized by a female to male difference in EAE (the C57BL/6), a sex chromosome effect was not present. The presence of a sex chromosome effect in one strain, but not another, revealed an interaction between sex chromosome complement and genetic background. Notably, effects of sex hormones in EAE have also previously been shown to be dependent on genetic background [72] and some autosomal gene linkages to susceptibility to MS have been identified in one gender but not the other [73]. Thus, in the outbred human population, the genetic background of some, but not all, individuals may be permissive to sex chromosome or sex hormone effects. Notably, as overall there is a sex difference in many autoimmune diseases in humans, we hypothesize that relatively permissive genetic backgrounds are likely to be prevalent, not rare, in occurrence.

In light of the fact that lupus in humans is characterized by a 9:1 female preponderance and since it had previously shown that pristane induced lupus in SJL mice was characterized by greater susceptibility in females as compared to males [233], it was then determined whether the effect of sex chromosome complement observed in SJL mice with EAE was unique to this disease or was more pervasive across autoimmune diseases. Thus, the role of sex chromosome complement in pristane induced lupus was investigated. Ovariectomized female XX and XY^- ^SJL mice and castrated male XX*Sry *and XY^-^*Sry *mice were injected with pristane and monitored daily for signs of disease. By 26 weeks only 30.8% of XX mice had survived, 68.4% of XY^- ^mice had survived. This significantly greater disease severity in XX mice also occurred when comparing castrated male XX*Sry *mice with XY^-^*Sry*. Kidney pathology and levels of anti-dsDNA antibodies also revealed more severe disease in XX as compared to XY^- ^mice [231].

Together, data using the informative *Sry *transgenic mice indicated that the XX sex chromosome complement was disease promoting as compared to the XY^- ^complement. It remains to be determined whether this effect is due to: (1) gene(s) unique to the Y chromosome; (2) a higher dose of X genes which escape X-inactivation in XX mice; or (3) paternal imprinting of X genes which are present in XX, but not XY^-^, mice [221]. Consistent with a potential X dosage effect in our lupus model, XXY men and XX women both have a similar high risk of developing lupus, while the incidence of lupus in XO females is very low [234,235]. Since these sex chromosome differences in humans are confounded by differences in sex hormones during both development and adulthood, it is difficult to distinguish between a sex chromosome effect versus a sex hormone effect in humans.

## Conclusions

The study of sex differences in autoimmune disease includes: (1) research addressing female versus male differences in a given autoimmune disease; and (2) research focused on a major disease modifier which is inherent to being one sex. In autoimmune diseases, there are two major clinical observations related to sex: first, females are more susceptible than males; and, second, pregnancy reduces relapses in females. Both of these observations can be pursued with respect to basic mechanism and translational potential.

With regard to the clinical observation that females are more susceptible than males, the state of being female indeed confers more susceptibility risk to develop autoimmune disease than any gene or environmental factor identified to date. Basic research indicates a protective role for high levels of androgens in young males, with little role for fluctuating levels of ovarian hormones in females. Thus clinical trials of treatment with androgens in autoimmune disease are warranted. Further, a role for sex hormones is not mutually exclusive of a role for sex chromosomes. Indeed, the XX sex chromosome complement has been shown to be disease promoting as compared to XY^-^. This sex chromosome effect is true in autoimmune disease models that are distinct in immunopathogenesis, including both cell mediated and antibody mediated diseases. The precise gene on X or Y remains to be determined.

With regard to the clinical observation that late pregnancy in females with MS is associated with an 80% reduction in relapses, this reduction is more dramatic than any of the first line drugs currently available to treat MS which reduce relapses by 30%-50%. A protective role for high levels of estrogens during late pregnancy has been shown in animal models. This is true for cell mediated, but not antibody mediated, diseases. Thus, clinical trials of treatment with pregnancy level oestrogens in cell mediated autoimmune disease are warranted.

Thus, the study of clinically relevant sex differences, if understood at the basic science level, can lead to new treatments for a variety of autoimmune diseases.

## Abbreviations

CNS: central nervous system; DHT: dihydrotesterone; EAE: experimental autoimmune encephalomyelitis (a mouse model of MS); ER: oestrogen receptor; HPA: hypothalamic-pituitary axis; IFN: interferon; IL: interleukin; MBP: myelin basic protein; MOG: myelin oligodendrocyte protein; MRI: magnetic resonance imaging; MS: multiple sclerosis; NAWN: normal appearing white matter; PLP: proteolipid protein; PBMC: peripheral blood lymphocytes; RA: rheumatoid arthritis; RR: relapsing remitting; SP: secondary progression; TNF: tumour necrosis factor; Th1: T helper 1;

## Glossary

25' timed walk = a measure of gait speed used as part of the MS Functional Composite Measure. The 25 foot timed walk is a mobility and leg function performance test in which MS patients are timed to walk 25 ft. Those patients unable to complete the 25 ft walk, need assistance or use a wheel chair, are noted as such.

Active EAE = EAE induced directly by immunization with an emulsion of adjuvant and stimulatory protein (autoantigen). This approach enables the study of disease inductor and effector phases of experimental demyelinating disease.

Adoptive EAE = CD4 T lymphocytes specific for the autoantigen are transferred from an immunized donor mouse to a naive recipient mouse, with disease then occurring in the recipient mouse. This method of EAE allows for the separation of the induction and effector phases of the disease.

Allograft = a transplant of tissue, cells, or organs from a genetically non-identical source of the same species.

Aromatase = an enzyme of the cytochrome P450 superfamily, which functions to aromatize androgens to produce oestrogens.

Diffusion-weighted imaging = a type of MRI in which *in vivo *images of biological tissue are weighted with the microstructural characteristics of water diffusion within the tissue.

Gadolinium enhancing lesions = a delineated area of increased MR signal intensity on T1-weighted spin-echo images, compared to normal-appearing surrounding brain tissue following intravenous injection of a gadolinium chelate.

Expanded Disability Status Scale (EDSS) = a method for quantifying disability in MS patients, in which neurologists assess eight functional systems: pyramidal, cerebellar, brainstem, sensory, bowel and bladder, visual, cerebral and other.

Hashimoto's thyroiditis = a type of autoimmune thyroid disease in which the immune system attacks and destroys the thyroid gland, causing a dysfunctional production of thyroid hormones.

Lymph node cells (LNCs) = cells cultured from the lymph nodes, organs belonging to the lymphatic system and found throughout the body. The lymph nodes house several immune cell types including B cells, T cells, plasma cells, macrophages, reticular cells and white blood cells.

Nine-hole peg task = the nine-hole peg task is used to measure fine manual dexterity of the right and left hands in MS patients.

Non-obese diabetic (NOD) mice = a mouse strain susceptible to spontaneous development of autoimmune insulin dependent diabetes mellitus.

Normal appearing white matter (NAWM) = myelinated area of the CNS that appears intact and healthy via MRI but that may contain abnormalities and lesions.

Oophorectomy = another term for ovariectomy, the surgical removal of the female reproductive organs, ovaries.

Paced Auditory Serial Addition Task (PASAT) = a cognitive task given to MS patients to measure auditory information processing speed and flexibility, as well as calculation ability.

Primary biliary cirrhosis = an autoimmune disease of the liver, in which the small bile ducts are slowly and progressively destroyed. This causes bile accumulation, which damages surrounding tissue and can lead to scarring, fibrosis, cirrhosis and eventual liver failure.

Pristane = a natural saturated alkaline found primarily in shark liver oil. Pristane is used as an immunologic adjuvant to induce systemic lupus erythematosus (SLE).

PD-1 = programmed death marker 1.

Sedimentation rate = also referred to as erythrocyte sedimentation rate (ESR), the speed at which red blood cells (erythrocytes) fall to the bottom of a fine glass tube filled with a RA patient's blood. A faster ESR indicates a higher level of inflammation, and aids in determining the severity of the condition.

Sertoli cells = a type of supporting cell found in the seminiferous tubules within the testes, important for maturation of spermatocytes.

Sjogren's syndrome = a chronic autoimmune disease in which white blood cells attack systemic moisture producing glands, such as the tear duct and salivation glands.

T2 lesion = a hyperintense region in an MRI scan, where normal appearing white matter would be dark.

T2-weighted imaging = a type of MRI contrast, in which water concentration affects image intensity. As an example, oedema would appear as a hyperintense spot on the MRI scan and white matter would appear dark in this contrast type.

Uterine endometrial hyperplasia = a condition in which the endometrium lining of the uterus excessively proliferates.

Wallerian degeneration = a process that results from the cutting or crushing of a nerve fibre in which the axon degenerates after it is detached from its neuronal cell body.

## Competing interests

UCLA holds a use patent for oestriol treatment in MS of which Dr Voskuhl is an inventor. Dr Voskuhl is a consultant for Adeona Pharmaceuticals receiving a maximum of $10,000 per year.

## Authors' contributions

RRV reviewed the literature and wrote the review.

## Author's information

RRV is a Professor of Neurology and Director of the UCLA Multiple Sclerosis Program. Her career has focused principally on basic science and clinical trials in multiple sclerosis.

## References

[B1] Calabresi PA Stone LA Bash CN Frank JA McFarland HF Interferon beta results in immediate reduction of contrast-enhanced MRI lesions in multiple sclerosis patients followed by weekly MRI Neurology 1997 48 5 1446 1448 9153489 10.1212/wnl.48.5.1446

[B2] Ge Y Grossman RI Udupa JK Fulton J Constantinescu CS Gonzales-Scarano F Babb JS Mannon LJ Kolson DL Cohen JA Glatiramer acetate (Copaxone) treatment in relapsing-remitting MS: quantitative MR assessment Neurology 2000 54 4 813 817 10690968 10.1212/wnl.54.4.813

[B3] Li DK Paty DW Magnetic resonance imaging results of the PRISMS trial: a randomized, double-blind, placebo-controlled study of interferon-beta1a in relapsing-remitting multiple sclerosis. Prevention of relapses and disability by interferon-beta1a subcutaneously in multiple sclerosis Ann Neurol 1999 46 2 197 206 10.1002/1531-8249(199908)46:2<197::AID-ANA9>3.0.CO;2-P 10443885

[B4] Simon JH Jacobs LD Campion M Wende K Simonian N Cookfair DL Rudick RA Herndon RM Richert JR Salazar AM Magnetic resonance studies of intramuscular interferon beta-1a for relapsing multiple sclerosis. The Multiple Sclerosis Collaborative Research Group [see comments] Ann Neurol 1998 43 1 79 87 10.1002/ana.410430114 9450771

[B5] Zhao GJ Koopmans RA Li DK Bedell L Paty DW Effect of interferon beta-1b in MS: assessment of annual accumulation of PD/T2 activity on MRI. UBC MS/MRI Analysis Group and the MS Study Group Neurology 2000 54 1 200 206 10636148 10.1212/wnl.54.1.200

[B6] Stevenson VL Miller DH Leary SM Rovaris M Barkhof F Brochet B Dousset V Filippi M Hintzen R Montalban X One year follow up study of primary and transitional progressive multiple sclerosis J Neurol Neurosurg Psychiatry 2000 68 6 713 718 10.1136/jnnp.68.6.713 10811693 PMC1736970

[B7] Rice GP Filippi M Comi G Cladribine and progressive MS: clinical and MRI outcomes of a multicenter controlled trial. Cladribine MRI Study Group Neurology 2000 54 5 1145 1155 10720289 10.1212/wnl.54.5.1145

[B8] Martin R McFarland HF McFarlin DE Immunological aspects of demyelinating diseases Ann Rev Immunol 1992 10 5 153 187 10.1146/annurev.iy.10.040192.001101 1375472

[B9] Waxman SG Demyelinating diseases--new pathological insights, new therapeutic targets [editorial; comment] NEJM 1998 338 5 323 325 9445415 10.1056/NEJM199801293380610

[B10] Wegmann TG Lin H Guilbert L Mosmann TR Bidirectional cytokine interactions in the maternal-fetal relationship: is successful pregnancy a TH2 phenomenon? [see comments] Immunol Today 1993 14 7 353 356 10.1016/0167-5699(93)90235-D 8363725

[B11] Aharoni R Teitelbaum D Sela M Arnon R Bystander suppression of experimental autoimmune encephalomyelitis by T cell lines and clones of the Th2 type induced by copolymer 1 J Neuroimmunol 1998 91 1-2 135 146 10.1016/S0165-5728(98)00166-0 9846830

[B12] Duda PW Schmied MC Cook SL Krieger JI Hafler DA Glatiramer acetate (Copaxone) induces degenerate, Th2-polarized immune responses in patients with multiple sclerosis J Clin Invest 2000 105 7 967 976 10.1172/JCI8970 10749576 PMC377485

[B13] Kozovska ME Hong J Zang YC Li S Rivera VM Killian JM Zhang JZ Interferon beta induces T-helper 2 immune deviation in MS Neurology 1999 53 8 1692 1697 10563614 10.1212/wnl.53.8.1692

[B14] Rudick RA Ransohoff RM Lee JC Peppler R Yu M Mathisen PM Tuohy VK *In vivo *effects of interferon beta-1a on immunosuppressive cytokines in multiple sclerosis [published erratum appears in Neurology 1998 Jul; 51(1): 332] Neurology 1998 50 5 1294 1300 9595977 10.1212/wnl.50.5.1294

[B15] Polman CH O'Connor PW Havrdova E Hutchinson M Kappos L Miller DH Phillips JT Lublin FD Giovannoni G Wajgt A A randomized, placebo-controlled trial of natalizumab for relapsing multiple sclerosis NEJM 2006 354 9 899 910 10.1056/NEJMoa044397 16510744

[B16] Coles AJ Cox A Le Page E Jones J Trip SA Deans J Seaman S Miller DH Hale G Waldmann H The window of therapeutic opportunity in multiple sclerosis: evidence from monoclonal antibody therapy J Neurol 2006 253 1 98 108 10.1007/s00415-005-0934-5 16044212

[B17] Brex PA Jenkins R Fox NC Crum WR O'Riordan JI Plant GT Miller DH Detection of ventricular enlargement in patients at the earliest clinical stage of MS Neurology 2000 54 8 1689 1691 10762518 10.1212/wnl.54.8.1689

[B18] Filippi M Bozzali M Rovaris M Gonen O Kesavadas C Ghezzi A Martinelli V Grossman RI Scotti G Comi G Evidence for widespread axonal damage at the earliest clinical stage of multiple sclerosis Brain 2003 126 Pt 2 433 437 10.1093/brain/awg038 12538409

[B19] Ge Y Grossman RI Udupa JK Wei L Mannon LJ Polansky M Kolson DL Brain atrophy in relapsing-remitting multiple sclerosis and secondary progressive multiple sclerosis: longitudinal quantitative analysis Radiology 2000 214 3 665 670 10715027 10.1148/radiology.214.3.r00mr30665

[B20] Losseff NA Wang L Lai HM Yoo DS Gawne CM McDonald WI Miller DH Thompson AJ Progressive cerebral atrophy in multiple sclerosis. A serial MRI study Brain 1996 2009 2019 10.1093/brain/119.6.2009 9010005

[B21] Rudick RA Fisher E Lee JC Simon J Jacobs L Use of the brain parenchymal fraction to measure whole brain atrophy in relapsing-remitting MS. Multiple Sclerosis Collaborative Research Group Neurology 1999 53 8 1698 1704 10563615 10.1212/wnl.53.8.1698

[B22] Stevenson VL Leary SM Losseff NA Parker GJ Barker GJ Husmani Y Miller DH Thompson AJ Spinal cord atrophy and disability in MS: a longitudinal study Neurology 1998 51 1 234 238 9674808 10.1212/wnl.51.1.234

[B23] Catalaa I Fulton JC Zhang X Udupa JK Kolson D Grossman M Wei L McGowan JC Polansky M Grossman RI MR imaging quantitation of gray matter involvement in multiple sclerosis and its correlation with disability measures and neurocognitive testing AJNR 1999 20 9 1613 1618 10543630 PMC7056175

[B24] Bakshi R Benedict RH Bermel RA Jacobs L Regional brain atrophy is associated with physical disability in multiple sclerosis: semiquantitative magnetic resonance imaging and relationship to clinical findings J Neuroimaging 2001 11 2 129 136 10.1111/j.1552-6569.2001.tb00022.x 11296581

[B25] Tortorella C Viti B Bozzali M Sormani MP Rizzo G Gilardi MF Comi G Filippi M A magnetization transfer histogram study of normal-appearing brain tissue in MS Neurology 2000 54 1 186 193 10636146 10.1212/wnl.54.1.186

[B26] Narayanan S Fu L Pioro E De Stefano N Collins DL Francis GS Antel JP Matthews PM Arnold DL Imaging of axonal damage in multiple sclerosis: spatial distribution of magnetic resonance imaging lesions Ann Neurol 1997 41 3 385 391 10.1002/ana.410410314 9066360

[B27] Gasperini C Horsfield MA Thorpe JW Kidd D Barker GJ Tofts PS MacManus DG Thompson AJ Miller DH McDonald WI Macroscopic and microscopic assessments of disease burden by MRI in multiple sclerosis: relationship to clinical parameters J Magn Reson Imaging 1996 6 4 580 584 10.1002/jmri.1880060404 8835949

[B28] Filippi M Iannucci G Cercignani M Assunta RM Pratesi A Comi G A quantitative study of water diffusion in multiple sclerosis lesions and normal-appearing white matter using echo-planar imaging Arch Neurol 2000 57 7 1017 1021 10.1001/archneur.57.7.1017 10891984

[B29] Filippi M Inglese M Rovaris M Sormani MP Horsfield P Iannucci PG Colombo B Comi G Magnetization transfer imaging to monitor the evolution of MS: a 1-year follow-up study [In Process Citation] Neurology 2000 55 7 940 946 11061248 10.1212/wnl.55.7.940

[B30] De Stefano N Narayanan S Matthews PM Francis GS Antel JP Arnold DL *In vivo *evidence for axonal dysfunction remote from focal cerebral demyelination of the type seen in multiple sclerosis Brain 1999 122 Pt 10 1933 1939 10.1093/brain/122.10.1933 10506094

[B31] Catalaa I Grossman RI Kolson DL Udupa JK Nyul LG Wei L Zhang X Polansky M Mannon LJ McGowan JC Multiple sclerosis: magnetization transfer histogram analysis of segmented normal-appearing white matter Radiology 2000 216 2 351 355 10924552 10.1148/radiology.216.2.r00au16351

[B32] Santos AC Narayanan S de Stefano N Tartaglia MC Francis SJ Arnaoutelis R Caramanos Z Antel JP Pike GB Arnold DL Magnetization transfer can predict clinical evolution in patients with multiple sclerosis J Neurol 2002 249 6 662 668 10.1007/s00415-002-0686-4 12111296

[B33] Peterson JW Bo L Mork S Chang A Trapp BD Transected neurites, apoptotic neurons, and reduced inflammation in cortical multiple sclerosis lesions Ann Neurol 2001 50 3 389 400 10.1002/ana.1123 11558796

[B34] Bo L Vedeler CA Nyland HI Trapp BD Mork SJ Subpial demyelination in the cerebral cortex of multiple sclerosis patients J Neuropathol Exp Neurol 2003 62 7 723 732 12901699 10.1093/jnen/62.7.723

[B35] Whitacre CC Reingold SC O'Looney PA A gender gap in autoimmunity Science 1999 283 5406 1277 1278 10.1126/science.283.5406.1277 10084932

[B36] Weinshenker BG Natural history of multiple sclerosis Ann Neurol 1994 36 Suppl S6 S11 10.1002/ana.410360704 8017890

[B37] Duquette P Pleines J Girard M Charest L Senecal-Quevillon M Masse C The increased susceptibility of women to multiple sclerosis Canadian J Neurolog Sci 1992 19 4 466 471 1423044

[B38] Orton SM Herrera BM Yee IM Valdar W Ramagopalan SV Sadovnick AD Ebers GC Sex ratio of multiple sclerosis in Canada: a longitudinal study Lancet Neurol 2006 5 11 932 936 10.1016/S1474-4422(06)70581-6 17052660

[B39] Libert C Dejager L Pinheiro I The X chromosome in immune functions: when a chromosome makes the difference Nat Rev Immunol 10 8 594 604 10.1038/nri2815 20651746

[B40] Butterworth M McClellan B Allansmith M Influence of sex in immunoglobulin levels Nature 1967 214 94 1224 1225 10.1038/2141224a0 4169229

[B41] Kantarci OH Hebrink DD Schaefer-Klein J Sun Y Achenbach S Atkinson EJ Heggarty S Cotleur AC de Andrade M Vandenbroeck K Interferon gamma allelic variants: sex-biased multiple sclerosis susceptibility and gene expression Arch Neurol 2008 65 3 349 357 10.1001/archneurol.2007.66 18332247

[B42] Moldovan IR Cotleur AC Zamor N Butler RS Pelfrey CM Multiple sclerosis patients show sexual dimorphism in cytokine responses to myelin antigens J Neuroimmunol 2008 193 1-2 161 169 10.1016/j.jneuroim.2007.10.010 18022700 PMC2235927

[B43] Pelfrey CM Cotleur AC Lee JC Rudick RA Sex differences in cytokine responses to myelin peptides in multiple sclerosis J Neuroimmunol 2002 130 1-2 211 223 10.1016/S0165-5728(02)00224-2 12225904

[B44] Confavreux C Vukusic S Adeleine P Early clinical predictors and progression of irreversible disability in multiple sclerosis: an amnesic process Brain 2003 126 Pt 4 770 782 10.1093/brain/awg081 12615637

[B45] Hawkins SA McDonnell GV Benign multiple sclerosis? Clinical course, long term follow up, and assessment of prognostic factors J Neurol Neurosurg Psychiatry 1999 67 2 148 152 10.1136/jnnp.67.2.148 10406979 PMC1736487

[B46] Pozzilli C Tomassini V Marinelli F Paolillo A Gasperini C Bastianello S 'Gender gap' in multiple sclerosis: magnetic resonance imaging evidence Eur J Neurol 2003 10 1 95 97 10.1046/j.1468-1331.2003.00519.x 12535003

[B47] Mokhtarian F McFarlin DE Raine CS Adoptive transfer of myelin basic protein-sensitized T cells produces chronic relapsing demyelinating disease in mice Nature 1984 309 5966 356 358 10.1038/309356a0 6203039

[B48] Brown A McFarlin DE Raine CS Chronologic neuropathology of relapsing experimental allergic encephalomyelitis in the mouse Lab Investigation 1982 46 2 171 185 7062721

[B49] Raine CS Cannella B Hauser SL Genain CP Demyelination in primate autoimmune encephalomyelitis and acute multiple sclerosis lesions: a case for antigen-specific antibody mediation Ann Neurol 1999 46 2 144 160 10.1002/1531-8249(199908)46:2<144::AID-ANA3>3.0.CO;2-K 10443879

[B50] Raine CS Barnett LB Brown A Behar T McFarlin DE Neuropathology of experimental allergic encephalomyelitis in inbred strains of mice Lab Investigation 1980 43 2 150 157 7401630

[B51] Wujek JR Bjartmar C Richer E Ransohoff RM Yu M Tuohy VK Trapp BD Axon loss in the spinal cord determines permanent neurological disability in an animal model of multiple sclerosis J Neuropathol Expl Neurol 2002 61 1 23 32 10.1093/jnen/61.1.23 11829341

[B52] Deboy CA Zhang J Dike S Shats I Jones M Reich DS Mori S Nguyen T Rothstein B Miller RH High resolution diffusion tensor imaging of axonal damage in focal inflammatory and demyelinating lesions in rat spinal cord Brain 2007 130 Pt 8 2199 21210 10.1093/brain/awm122 17557778

[B53] Morales LB Loo KK Liu HB Peterson C Tiwari-Woodruff S Voskuhl RR Treatment with an estrogen receptor alpha ligand is neuroprotective in experimental autoimmune encephalomyelitis J Neurosci 2006 26 25 6823 6833 10.1523/JNEUROSCI.0453-06.2006 16793889 PMC6673842

[B54] Tiwari-Woodruff S Morales LB Lee R Voskuhl RR Differential neuroprotective and antiinflammatory effects of estrogen receptor (ER){alpha} and ERbeta ligand treatment Proc Natl Acad Sci USA 2007 104 37 14813 14818 10.1073/pnas.0703783104 17785421 PMC1976208

[B55] Bannerman PG Hahn A Ramirez S Morley M Bonnemann C Yu S Zhang GX Rostami A Pleasure D Motor neuron pathology in experimental autoimmune encephalomyelitis: studies in THY1-YFP transgenic mice Brain 2005 128 Pt 8 1877 1886 10.1093/brain/awh550 15901645

[B56] Kim JH Budde MD Liang HF Klein RS Russell JH Cross AH Song SK Detecting axon damage in spinal cord from a mouse model of multiple sclerosis Neurobiol Dis 2006 21 3 626 632 10.1016/j.nbd.2005.09.009 16298135

[B57] Mackenzie-Graham A Tinsley MR Shah KP Aguilar C Strickland LV Boline J Martin M Morales L Shattuck DW Jacobs RE Cerebellar cortical atrophy in experimental autoimmune encephalomyelitis Neuroimage 2006 32 3 1016 23 10.1016/j.neuroimage.2006.05.006 16806982

[B58] Bebo BF Jr Adlard K Schuster JC Unsicker L Vandenbark AA Offner H Gender differences in protection from EAE induced by oral tolerance with a peptide analogue of MBP-Ac1-11 J Neurosci Res 1999 55 4 432 440 10.1002/(SICI)1097-4547(19990215)55:4<432::AID-JNR4>3.0.CO;2-2 10723054

[B59] Bebop BF Jar Schuster JC Vandenberg AA Offer H Gender differences in experimental autoimmune encephalomyelitis develop during the induction of the immune response to encephalitogenic peptides J Neurosci Res 1998 52 4 420 426 10.1002/(SICI)1097-4547(19980515)52:4<420::AID-JNR5>3.0.CO;2-B 9589386

[B60] Bebo BF Jr Vandenbark AA Offner H Male SJL mice do not relapse after induction of EAE with PLP 139-151 J Neurosci Res 1996 45 6 680 689 10.1002/(SICI)1097-4547(19960915)45:6<680::AID-JNR4>3.0.CO;2-4 8892079

[B61] Kim S Voskuhl RR Decreased IL-12 production underlies the decreased ability of male lymph node cells to induce experimental autoimmune encephalomyelitis J Immunol 1999 162 9 5561 5568 10228038

[B62] Voskuhl RR Pitchekian-Halabi H MacKenzie-Graham A McFarland HF Raine CS Gender differences in autoimmune demyelination in the mouse: implications for multiple sclerosis Ann Neurol 1996 39 6 724 733 10.1002/ana.410390608 8651644

[B63] Bebop BF Jar Delink-Vincent E Adams G Amundsen D Vandenberg AA Offer H Gonad hormones influence the immune response to PLP 139-151 and the clinical course of relapsing experimental autoimmune encephalomyelitis J Neuroimmunol 1998 84 2 122 130 10.1016/S0165-5728(97)00214-2 9628453

[B64] Smith ME Eller NL McFarland HF Racke MK Raine CS Age dependence of clinical and pathological manifestations of autoimmune demyelination. Implications for multiple sclerosis Am J Pathol 1999 155 4 1147 1161 10514398 10.1016/S0002-9440(10)65218-2 PMC1867019

[B65] Fitzpatrick F Lepault F Homo-Delarche F Bach JF Dardenne M Influence of castration, alone or combined with thymectomy, on the development of diabetes in the nonobese diabetic mouse Endocrinology 1991 129 3 1382 1390 10.1210/endo-129-3-1382 1874178

[B66] Ahmed SA Penhale WJ The influence of testosterone on the development of autoimmune thyroiditis in thymectomized and irradiated rats Clin Expl Immunol 1982 48 2 367 374 PMC1536459 7049452

[B67] Fox HS Androgen treatment prevents diabetes in nonobese diabetic mice J Expl Med 1992 175 5 1409 1412 10.1084/jem.175.5.1409 PMC2119211 1569406

[B68] Harbuz MS Perveen-Gill Z Lightman SL Jessop DS A protective role for testosterone in adjuvant-induced arthritis Br J Rheumatol 1995 34 12 1117 1122 10.1093/rheumatology/34.12.1117 8608351

[B69] Foster SC Daniels C Bourdette DN Bebo BF Jr Dysregulation of the hypothalamic-pituitary-gonadal axis in experimental autoimmune encephalomyelitis and multiple sclerosis J Neuroimmunol 2003 140 1-2 78 87 10.1016/S0165-5728(03)00177-2 12864974

[B70] Papenfuss TL Rogers CJ Gienapp I Yurrita M McClain M Damico N Valo J Song F Whitacre CC Sex differences in experimental autoimmune encephalomyelitis in multiple murine strains J Neuroimmunol 2004 150 1-2 59 69 10.1016/j.jneuroim.2004.01.018 15081249

[B71] Okuda Y Okuda M Bernard CC Gender does not influence the susceptibility of C57BL/6 mice to develop chronic experimental autoimmune encephalomyelitis induced by myelin oligodendrocyte glycoprotein Immunol Lett 2002 81 1 25 29 10.1016/S0165-2478(01)00339-X 11841842

[B72] Palaszynski KM Loo KK Ashouri JF Liu H Voskuhl RR Androgens are protective in experimental autoimmune encephalomyelitis: implications for multiple sclerosis J Neuroimmunol 2004 146 1-2 144 152 10.1016/j.jneuroim.2003.11.004 14698857

[B73] Kantarci OH Goris A Hebrink DD Heggarty S Cunningham S Alloza I Atkinson EJ de Andrade M McMurray CT Graham CA IFNG polymorphisms are associated with gender differences in susceptibility to multiple sclerosis Genes Immun 2005 6 153 161 10.1038/sj.gene.6364164 15674394

[B74] Spach KM Blake M Bunn JY McElvany B Noubade R Blankenhorn EP Teuscher C Cutting edge: the Y chromosome controls the age-dependent experimental allergic encephalomyelitis sexual dimorphism in SJL/J mice J Immunol 2009 182 4 1789 1793 10.4049/jimmunol.0803200 19201829 PMC2658649

[B75] Butterfield RJ Blankenhorn EP Roper RJ Zachary JF Doerge RW Sudweeks J Rose J Teuscher C Genetic analysis of disease subtypes and sexual dimorphisms in mouse experimental allergic encephalomyelitis (EAE): relapsing/remitting and monophasic remitting/nonrelapsing EAE are immunogenetically distinct J Immunol 1999 162 5 3096 3102 10072563

[B76] Jiang PP Frederick K Hansen TH Miller RD Localization of the mouse gene releasing sex-limited expression of Slp Proc Natl Acad Sci USA 1996 93 2 913 917 10.1073/pnas.93.2.913 8570659 PMC40158

[B77] Dalal M Kim S Voskuhl RR Testosterone therapy ameliorates experimental autoimmune encephalomyelitis and induces a T helper 2 bias in the autoantigen-specific T lymphocyte response J Immunol 1997 159 1 3 6 9200430

[B78] Liva SM Voskuhl RR Testosterone acts directly on CD4+ T-lymphocytes to increase IL10 production J Immunol 2001 167 2060 2067 11489988 10.4049/jimmunol.167.4.2060

[B79] Bebop BF Jar Schuster JC Vandenberg AA Offer H Androgens alter the cytokine profile and reduce encephalitogenicity of myelin-reactive T cells J Immunol 1999 162 1 35 40 9886367

[B80] Wilcoxen SC Kirkman E Dowdell KC Stohlman SA Gender-dependent IL-12 secretion by APC is regulated by IL-10 J Immunol 2000 164 12 6237 6243 10843676 10.4049/jimmunol.164.12.6237

[B81] Gray A Berlin JA McKinlay JB Longcope C An examination of research design effects on the association of testosterone and male aging: results of a meta-analysis J Clin Epidemiol 1991 44 7 671 684 10.1016/0895-4356(91)90028-8 1829756

[B82] Morley JE Kaiser FE Perry HM Patrick P Morley PM Stauber PM Vellas B Baumgartner RN Garry PJ Longitudinal changes in testosterone, luteinizing hormone, and follicle-stimulating hormone in healthy older men Metabolism Clin Expl 1997 46 4 410 413 10.1016/s0026-0495(97)90057-3 9109845

[B83] Nankin HR Calkins JH Decreased bioavailable testosterone in aging normal and impotent men J Clin Endocrinol Metabolism 1986 63 6 1418 1420 10.1210/jcem-63-6-1418 3782425

[B84] Tenover JS Matsumoto AM Plymate SR Bremner WJ The effects of aging in normal men on bioavailable testosterone and luteinizing hormone secretion: response to clomiphene citrate J Clin Endocrinol Metabolism 1987 65 6 1118 1126 10.1210/jcem-65-6-1118 3119649

[B85] Vermeulen A Clinical review 24: Androgens in the aging male J Clin Endocrinol Metabolism 1991 73 2 221 224 10.1210/jcem-73-2-221 1856256

[B86] Doran MF Pond GR Crowson CS O'Fallon M Gabriel SE Trends in Incidence and mortality in rheumatoid arthritis in Rochester, Minnesota, over a forty-year period Arthritis Rheumatism 2002 46 3 625 631 10.1002/art.509 11920397

[B87] Wei T Lightman SL The neuroendocrine axis in patients with multiple sclerosis Brain 1997 120 Pt 6; 5406 1067 1076 10.1093/brain/120.6.1067 9217689

[B88] Kanik KS Chrousos GP Schumacher HR Crane ML Yarboro CH Wilder RL Adrenocorticotropin, glucocorticoid, and androgen secretion in patients with new onset synovitis/rheumatoid arthritis: relations with indices of inflammation J Clin Endocrinol Metab 2000 85 4 1461 1466 10.1210/jc.85.4.1461 10770182

[B89] Sicotte NL Giesser BS Tandon V Klutch R Steiner B Drain AE Shattuck DW Hull L Wang HJ Elashoff RM Testosterone treatment in multiple sclerosis: a pilot study Arch Neurol 2007 64 5 683 688 10.1001/archneur.64.5.683 17502467

[B90] Stone LA Frank JA Albert PS Bash CN Calabresi PA Maloni H McFarland HF Characterization of MRI response to treatment with interferon beta-1b: contrast-enhancing MRI lesion frequency as a primary outcome measure Neurology 1997 49 3 862 869 9305355 10.1212/wnl.49.3.862

[B91] Janowsky JS Chavez B Orwoll E Sex steroids modify working memory J Cogn Neurosci 2000 12 3 407 414 10.1162/089892900562228 10931767

[B92] Cherrier MM Asthana S Plymate S Baker L Matsumoto AM Peskind E Raskind MA Brodkin K Bremner W Petrova A Testosterone supplementation improves spatial and verbal memory in healthy older men Neurology 2001 57 1 80 88 11445632 10.1212/wnl.57.1.80

[B93] Gold SM Chalifoux S Giesser BS Voskuhl RR Immune modulation and increased neurotrophic factor production in multiple sclerosis patients treated with testosterone J Neuroinflammation 2008 5 32 10.1186/1742-2094-5-32 18671877 PMC2518142

[B94] Abramsky O Pregnancy and multiple sclerosis Ann Neurol 1994 36 Suppl 1 S38 S41 10.1002/ana.410360712 7517125

[B95] Birk K Ford C Smeltzer S Ryan D Miller R Rudick RA The clinical course of multiple sclerosis during pregnancy and the puerperium Arch Neurol 1990 47 7 738 742 1972617 10.1001/archneur.1990.00530070026007

[B96] Confavreux C Hutchinson M Hours MM Cortinovis-Tourniaire P Moreau T Rate of pregnancy-related relapse in multiple sclerosis. Pregnancy in Multiple Sclerosis Group [see comments] NE J M 1998 339 5 285 291 10.1056/NEJM199807303390501 9682040

[B97] Da Silva JA Spector TD The role of pregnancy in the course and aetiology of rheumatoid arthritis Clin Rheumatol 1992 11 2 189 194 10.1007/BF02207955 1617891

[B98] Damek DM Shuster EA Pregnancy and multiple sclerosis Mayo Clinic Proc 1997 72 10 977 989 10.4065/72.10.977 9379704

[B99] Nelson JL Hughes KA Smith AG Nisperos BB Branchaud AM Hansen JA Remission of rheumatoid arthritis during pregnancy and maternal-fetal class II alloantigen disparity Am J Reproductive Immunol 1992 28 3-4 226 227 10.1111/j.1600-0897.1992.tb00798.x 1285885

[B100] Runmarker B Andersen O Pregnancy is associated with a lower risk of onset and a better prognosis in multiple sclerosis [see comments] Brain 1995 118 Pt 1: 10 253 261 10.1093/brain/118.1.253 7895009

[B101] Jungers P Dougados M Pélissier C Kuttenn F Tron F Lesavre P Bach JF Lupus nephropathy and pregnancy. Report of 104 cases in 36 patients Arch Intern Med 1982 142 4 771 776 10.1001/archinte.142.4.771 7073417

[B102] van Walderveen MA Tas MW Barkhof F Polman CH Frequin ST Hommes OR Valk J Magnetic resonance evaluation of disease activity during pregnancy in multiple sclerosis Neurology 1994 44 2 327 329 8309584 10.1212/wnl.44.2.327

[B103] Formby B Immunologic response in pregnancy. Its role in endocrine disorders of pregnancy and influence on the course of maternal autoimmune diseases Endocrinol Metabolism Clin N Am 1995 24 1 187 205 7781626

[B104] Hill JA Polgar K Anderson DJ T-helper 1-type immunity to trophoblast in women with recurrent spontaneous abortion [see comments] JAMA 1995 273 24 1933 1936 10.1001/jama.273.24.1933 7783303

[B105] Raghupathy R Th1-type immunity is incompatible with successful pregnancy [see comments] Immunol Today 1997 18 10 478 482 10.1016/S0167-5699(97)01127-4 9357139

[B106] Krishnan L Guilbert LJ Wegmann TG Belosevic M Mosmann TR T helper 1 response against Leishmania major in pregnant C57BL/6 mice increases implantation failure and fetal resorptions. Correlation with increased IFN-gamma and TNF and reduced IL-10 production by placental cells J Immunol 1996 156 2 653 662 8543817

[B107] Marzi M Vigano A Trabattoni D Villa ML Salvaggio A Clerici E Clerici M Characterization of type 1 and type 2 cytokine production profile in physiologic and pathologic human pregnancy Clin Expl Immunol 1996 106 1 127 133 10.1046/j.1365-2249.1996.d01-809.x PMC2200555 8870710

[B108] Lin H Mosmann TR Guilbert L Tuntipopipat S Wegmann TG Synthesis of T helper 2-type cytokines at the maternal-fetal interface J Immunol 1993 151 9 4562 4573 8409418

[B109] Sacks GP Clover LM Bainbridge DR Redman CW Sargent IL Flow cytometric measurement of intracellular Th1 and Th2 cytokine production by human villous and extravillous cytotrophoblast Placenta 2001 22 6 550 559 10.1053/plac.2001.0686 11440543

[B110] Dudley DJ Chen CL Mitchell MD Daynes RA Araneo BA Adaptive immune responses during murine pregnancy: pregnancy-induced regulation of lymphokine production by activated T lymphocytes Am J Obstet Gynecol 1993 168 4 1155 1163 8475961 10.1016/0002-9378(93)90361-l

[B111] Elenkov IJ Wilder RL Bakalov VK Link AA Dimitrov MA Fisher S Crane M Kanik KS Chrousos GP IL-12, TNF-alpha, and hormonal changes during late pregnancy and early postpartum: implications for autoimmune disease activity during these times J Clin Endocrinol Metab 2001 86 10 4933 4938 10.1210/jc.86.10.4933 11600565

[B112] Fabris N Piantanelli L Muzzioli M Differential effect of pregnancy or gestagens on humoral and cell-mediated immunity Clin Expl Immunol 1977 28 2 306 314 PMC1540744 141352

[B113] Gilmore W Arias M Stroud N Stek A McCarthy KA Correale J Preliminary studies of cytokine secretion patterns associated with pregnancy in MS patients J Neurol Sci 2004 224 1-2 69 76 10.1016/j.jns.2004.06.011 15450773

[B114] Al-Shammri S Rawoot P Azizieh F AbuQoora A Hanna M Saminathan TR Raghupathy R Th1/Th2 cytokine patterns and clinical profiles during and after pregnancy in women with multiple sclerosis J Neurol Sci 2004 222 1-2 21 27 10.1016/j.jns.2004.03.027 15240191

[B115] Voskuhl R Cohen D, Rudick R Sex hormomes and other pregnancy-related factors with therapeutic potential in multiple sclerosis Multiple Sclerosis Therapeutics 2003 32 2 London: Martin Dunitz 535 549

[B116] Gregg C Shikar V Larsen P Mak G Chojnacki A Yong VW Weiss S White matter plasticity and enhanced remyelination in the maternal CNS J Neurosci 2007 27 8 1812 1823 10.1523/JNEUROSCI.4441-06.2007 17314279 PMC6673564

[B117] Riskind PN Massacesi L Doolittle TH Hauser SL The role of prolactin in autoimmune demyelination: suppression of experimental allergic encephalomyelitis by bromocriptine Ann Neurol 1991 29 5 542 547 10.1002/ana.410290514 1859183

[B118] Vukusic S Hutchinson M Hours M Moreau T Cortinovis-Tourniaire P Adeleine P Confavreux C The Pregnancy In Multiple Sclerosis G Pregnancy and multiple sclerosis (the PRIMS study): clinical predictors of post-partum relapse Brain 2004 127 Pt 6 1353 1360 10.1093/brain/awh152 15130950

[B119] Craig A Ling Luo N Beardsley DJ Wingate-Pearse N Walker DW Hohimer AR Back SA Quantitative analysis of perinatal rodent oligodendrocyte lineage progression and its correlation with human Exp Neurol 2003 181 2 231 240 10.1016/S0014-4886(03)00032-3 12781996

[B120] Langer-Gould A Garren H Slansky A Ruiz PJ Steinman L Late pregnancy suppresses relapses in experimental autoimmune encephalomyelitis: evidence for a suppressive pregnancy-related serum factor J Immunol 2002 169 2 1084 1091 12097417 10.4049/jimmunol.169.2.1084

[B121] Voskuhl RR Palaszynski K Sex hormones and experimental autoimmune encephalomyelitis: implications for multiple sclerosis The Neuroscientist 2001 7 3 258 270 10.1177/107385840100700310 11499404

[B122] Bebo BF Jr Fyfe-Johnson A Adlard K Beam AG Vandenbark AA Offner H Low-dose estrogen therapy ameliorates experimental autoimmune encephalomyelitis in two different inbred mouse strains J Immunol 2001 166 2080 2089 11160259 10.4049/jimmunol.166.3.2080

[B123] Ito A Bebo BF Jr Matejuk A Zamora A Silverman M Fyfe-Johnson A Offner H Estrogen treatment down-regulates TNF-alpha production and reduces the severity of experimental autoimmune encephalomyelitis in cytokine knockout mice J Immunol 2001 167 1 542 552 11418693 10.4049/jimmunol.167.1.542

[B124] Liu HY Buenafe AC Matejuk A Ito A Zamora A Dwyer J Vandenbark AA Offner H Estrogen inhibition of EAE involves effects on dendritic cell function J Neurosci Res 2002 70 2 238 248 10.1002/jnr.10409 12271473

[B125] Matejuk A Adlard K Zamora A Silverman M Vandenbark AA Offner H 17beta-estradiol inhibits cytokine, chemokine and chemokine receptor mRNA expression in the central nervous system of female mice with experimental autoimmune encephalomyelitis J Neurosci Res 2001 65 6 529 542 10.1002/jnr.1183 11550221

[B126] Polanczyk M Zamora A Subramanian S Matejuk A Hess DL Blankenhorn EP Teuscher C Vandenbark AA Offner H The protective effect of 17beta-estradiol on experimental autoimmune encephalomyelitis is mediated through estrogen receptor-alpha Am J Pathol 2003 163 4 1599 1605 14507666 10.1016/s0002-9440(10)63516-x PMC1868300

[B127] Subramanian S Matejuk A Zamora A Vandenbark AA Offner H Oral feeding with ethinyl estradiol suppresses and treats experimental autoimmune encephalomyelitis in SJL mice and inhibits the recruitment of inflammatory cells into the central nervous system J Immunol 2003 170 3 1548 1555 12538720 10.4049/jimmunol.170.3.1548

[B128] Jansson L Olsson T Holmdahl R Estrogen induces a potent suppression of experimental autoimmune encephalomyelitis and collagen-induced arthritis in mice J Neuroimmunol 1994 53 2 203 207 10.1016/0165-5728(94)90030-2 8071434

[B129] Kim S Liva SM Dalal MA Verity MA Voskuhl RR Estriol ameliorates autoimmune demyelinating disease: implications for multiple sclerosis Neurology 1999 52 6 1230 1238 10214749 10.1212/wnl.52.6.1230

[B130] Liu HB Loo KK Palaszynski K Ashouri J Lubahn DB Voskuhl RR Estrogen receptor alpha mediates estrogen's immune protection in autoimmune disease J Immunol 2003 171 12 6936 6940 14662901 10.4049/jimmunol.171.12.6936

[B131] Palaszynski KM Liu H Loo KK Voskuhl RR Estriol treatment ameliorates disease in males with experimental autoimmune encephalomyelitis: implications for multiple sclerosis J Neuroimmunol 2004 149 1-2 84 89 10.1016/j.jneuroim.2003.12.015 15020068

[B132] Jansson L Holmdahl R Oestrogen-induced suppression of collagen arthritis; 17 beta-oestradiol is therapeutically active in normal and castrated F1 hybrid mice of both sexes Clin Expl Immunol 1992 89 3 446 451 10.1111/j.1365-2249.1992.tb06978.x PMC1554486 1516260

[B133] Jansson L Holmdahl R Estrogen-mediated immunosuppression in autoimmune diseases Inflammation Res 1998 47 7 290 301 10.1007/s000110050332 9719493

[B134] Jansson L Holmdahl R Enhancement of collagen-induced arthritis in female mice by estrogen receptor blockage Arthritis Rheum 2001 44 9 2168 2175 10.1002/1529-0131(200109)44:9<2168::AID-ART370>3.0.CO;2-2 11592382

[B135] Jansson L Mattsson A Mattsson R Holmdahl R Estrogen induced suppression of collagen arthritis. V: Physiological level of estrogen in DBA/1 mice is therapeutic on established arthritis, suppresses anti-type II collagen T-cell dependent immunity and stimulates polyclonal B-cell activity J Autoimmunity 1990 3 3 257 270 10.1016/0896-8411(90)90145-I 2397019

[B136] Melez KA Boegel WA Steinberg AD Therapeutic studies in New Zealand mice. VII. Successful androgen treatment of NZB/NZW F1 females of different ages Arthritis Rheum 1980 23 1 41 47 10.1002/art.1780230108 7352943

[B137] Shim GJ Kis LL Warner M Gustafsson JA Autoimmune glomerulonephritis with spontaneous formation of splenic germinal centers in mice lacking the estrogen receptor alpha gene Proc Natl Acad Sci USA 2004 101 6 1720 1724 10.1073/pnas.0307915100 14745006 PMC341834

[B138] Steinberg AD Melez KA Raveche ES Reeves JP Boegel WA Smathers PA Taurog JD Weinlein L Duvic M Approach to the study of the role of sex hormones in autoimmunity Arthritis Rheum 1979 22 11 1170 1176 10.1002/art.1780221103 508371

[B139] Bynoe MS Grimaldi CM Diamond B Estrogen up-regulates Bcl-2 and blocks tolerance induction of naive B cells Proc Natl Acad Sci USA 2000 97 6 2703 2708 10.1073/pnas.040577497 10694576 PMC15993

[B140] Grimaldi CM Cleary J Dagtas AS Moussai D Diamond B Estrogen alters thresholds for B cell apoptosis and activation J Clin Invest 2002 109 12 1625 1633 12070310 10.1172/JCI14873 PMC151010

[B141] Grimaldi CM Michael DJ Diamond B Cutting edge: expansion and activation of a population of autoreactive marginal zone B cells in a model of estrogen-induced lupus J Immunol 2001 167 4 1886 1890 11489967 10.4049/jimmunol.167.4.1886

[B142] Vidaver R Molecular and clinical evidence of the role of estrogen in lupus Trends Immunol 2002 23 5 229 230 10.1016/S1471-4906(02)02204-4 12102733

[B143] Gago N Akwa Y Sananes N Guennoun R Baulieu EE El-Etr M Schumacher M Progesterone and the oligodendroglial lineage: stage-dependent biosynthesis and metabolism Glia 2001 36 3 295 308 10.1002/glia.1117 11746767

[B144] Baulieu E Schumacher M Progesterone as a neuroactive neurosteroid, with special reference to the effect of progesterone on myelination Steroids 2000 65 10-11 605 612 10.1016/S0039-128X(00)00173-2 11108866

[B145] Schumacher M Guennoun R Robert F Carelli C Gago N Ghoumari A Gonzalez Deniselle MC Gonzalez SL Ibanez C Labombarda F Local synthesis and dual actions of progesterone in the nervous system: neuroprotection and myelination Growth Horm IGF Res 2004 14 Suppl A S18 33 10.1016/j.ghir.2004.03.007 15135772

[B146] Ghoumari AM Ibanez C El-Etr M Leclerc P Eychenne B O'Malley BW Baulieu EE Schumacher M Progesterone and its metabolites increase myelin basic protein expression in organotypic slice cultures of rat cerebellum J Neurochem 2003 86 4 848 859 10.1046/j.1471-4159.2003.01881.x 12887683

[B147] Hoffman GE Le WW Murphy AZ Koski CL Divergent effects of ovarian steroids on neuronal survival during experimental allergic encephalitis in Lewis rats Expl Neurol 2001 171 2 272 284 10.1006/exnr.2001.7783 11573979

[B148] Jansson L Holmdahl R Oestrogen induced suppression of collagen arthritis. IV: Progesterone alone does not affect the course of arthritis but enhances the oestrogen-mediated therapeutic effect J Reproductive Immunol 1989 15 2 141 150 10.1016/0165-0378(89)90033-8 2769647

[B149] Wang C Dehghani B Li Y Kaler LJ Vandenbark AA Offner H Oestrogen modulates experimental autoimmune encephalomyelitis and interleukin-17 production via programmed death 1 Immunology 2009 126 3 329 335 10.1111/j.1365-2567.2008.03051.x 19302141 PMC2669813

[B150] Zhang QH Hu YZ Cao J Zhong YQ Zhao YF Mei QB Estrogen influences the differentiation, maturation and function of dendritic cells in rats with experimental autoimmune encephalomyelitis Acta Pharmacol Sin 2004 25 4 508 513 15066222

[B151] Xiao BG Liu X Link H Antigen-specific T cell functions are suppressed over the estrogen-dendritic cell-indoleamine 2,3-dioxygenase axis Steroids 2004 69 10 653 659 10.1016/j.steroids.2004.05.019 15465110

[B152] Munn DH Zhou M Attwood JT Bondarev I Conway SJ Marshall B Brown C Mellor AL Prevention of allogeneic fetal rejection by tryptophan catabolism Science 1998 281 5380 1191 1193 10.1126/science.281.5380.1191 9712583

[B153] Mellor AL Munn DH IDO expression by dendritic cells: tolerance and tryptophan catabolism Nat Rev Immunol 2004 4 10 762 774 10.1038/nri1457 15459668

[B154] Polanczyk MJ Carson BD Subramanian S Afentoulis M Vandenbark AA Ziegler SF Offner H Cutting edge: estrogen drives expansion of the CD4+CD25+ regulatory T cell compartment J Immunol 2004 173 4 2227 2230 15294932 10.4049/jimmunol.173.4.2227

[B155] Matejuk A Bakke AC Hopke C Dwyer J Vandenbark AA Offner H Estrogen treatment induces a novel population of regulatory cells, which suppresses experimental autoimmune encephalomyelitis J Neurosci Res 2004 77 1 119 126 10.1002/jnr.20145 15197745

[B156] Brinton RD Cellular and molecular mechanisms of estrogen regulation of memory function and neuroprotection against Alzheimer's disease: recent insights and remaining challenges Learn Mem 2001 8 3 121 133 10.1101/lm.39601 11390632

[B157] Garcia-Segura LM Azcoitia I DonCarlos LL Neuroprotection by estradiol Prog Neurobiol 2001 63 1 29 60 10.1016/S0301-0082(00)00025-3 11040417

[B158] Wise PM Dubal DB Wilson ME Rau SW Bottner M Minireview: neuroprotective effects of estrogen-new insights into mechanisms of action Endocrinology 2001 142 3 969 973 10.1210/en.142.3.969 11181507

[B159] Sribnick EA Wingrave JM Matzelle DD Ray SK Banik NL Estrogen as a neuroprotective agent in the treatment of spinal cord injury Ann N Y Acad Sci 2003 993 125 133 discussion 159-160 10.1111/j.1749-6632.2003.tb07521.x 12853305

[B160] Goodman Y Bruce AJ Cheng B Mattson MP Estrogens attenuate and corticosterone exacerbates excitotoxicity, oxidative injury, and amyloid beta-peptide toxicity in hippocampal neurons J Neurochem 1996 66 5 1836 1844 10.1046/j.1471-4159.1996.66051836.x 8780008

[B161] Harms C Lautenschlager M Bergk A Katchanov J Freyer D Kapinya K Herwig U Megow D Dirnagl U Weber JR Differential mechanisms of neuroprotection by 17 beta-estradiol in apoptotic versus necrotic neurodegeneration J Neurosci 2001 21 8 2600 2609 11306613 10.1523/JNEUROSCI.21-08-02600.2001 PMC6762539

[B162] Behl C Skutella T Lezoualc'h F Post A Widmann M Newton CJ Holsboer F Neuroprotection against oxidative stress by estrogens: structure- activity relationship Molec Pharmacol 1997 51 4 535 541 9106616

[B163] Behl C Widmann M Trapp T Holsboer F 17-beta estradiol protects neurons from oxidative stress-induced cell death in vitro Biochem Biophys Res Commun 1995 216 2 473 482 10.1006/bbrc.1995.2647 7488136

[B164] Zhao L Wu TW Brinton RD Estrogen receptor subtypes alpha and beta contribute to neuroprotection and increased Bcl-2 expression in primary hippocampal neurons Brain Res 2004 1010 1-2 22 34 10.1016/j.brainres.2004.02.066 15126114

[B165] Sribnick EA Ray SK Nowak MW Li L Banik NL 17beta-estradiol attenuates glutamate-induced apoptosis and preserves electrophysiologic function in primary cortical neurons J Neurosci Res 2004 76 5 688 696 10.1002/jnr.20124 15139027

[B166] Bruce-Keller AJ Keeling JL Keller JN Huang FF Camondola S Mattson MP Antiinflammatory effects of estrogen on microglial activation Endocrinology 2000 141 10 3646 3656 10.1210/en.141.10.3646 11014219

[B167] Vegeto E Bonincontro C Pollio G Sala A Viappiani S Nardi F Brusadelli A Viviani B Ciana P Maggi A Estrogen prevents the lipopolysaccharide-induced inflammatory response in microglia J Neurosci 2001 21 6 1809 1818 11245665 10.1523/JNEUROSCI.21-06-01809.2001 PMC6762610

[B168] Drew PD Chavis JA Female sex steroids: effects upon microglial cell activation J Neuroimmunol 2000 111 1-2 77 85 10.1016/S0165-5728(00)00386-6 11063824

[B169] Garcia-Segura LM Naftolin F Hutchison JB Azcoitia I Chowen JA Role of astroglia in estrogen regulation of synaptic plasticity and brain repair J Neurobiol 1999 40 4 574 584 10.1002/(SICI)1097-4695(19990915)40:4<574::AID-NEU12>3.0.CO;2-8 10453057

[B170] Azcoitia I Sierra A Garcia-Segura LM Localization of estrogen receptor beta-immunoreactivity in astrocytes of the adult rat brain Glia 1999 26 3 260 267 10.1002/(SICI)1098-1136(199905)26:3<260::AID-GLIA7>3.0.CO;2-R 10340766

[B171] Sur P Sribnick EA Wingrave JM Nowak MW Ray SK Banik NL Estrogen attenuates oxidative stress-induced apoptosis in C6 glial cells Brain Res 2003 971 2 178 188 10.1016/S0006-8993(03)02349-7 12706234

[B172] Takao T Flint N Lee L Ying X Merrill J Chandross KJ 17beta-estradiol protects oligodendrocytes from cytotoxicity induced cell death J Neurochem 2004 89 3 660 673 10.1111/j.1471-4159.2004.02370.x 15086523

[B173] Cantarella G Risuglia N Lombardo G Lempereur L Nicoletti F Memo M Bernardini R Protective effects of estradiol on TRAIL-induced apoptosis in a human oligodendrocytic cell line: evidence for multiple sites of interactions Cell Death Differ 2004 11 503 311 10.1038/sj.cdd.4401367 14739940

[B174] Zhang Z Cerghet M Mullins C Williamson M Bessert D Skoff R Comparison of *in vivo *and *in vitro *subcellular localization of estrogen receptors alpha and beta in oligodendrocytes J Neurochem 2004 89 3 674 684 10.1111/j.1471-4159.2004.02388.x 15086524

[B175] Yune TY Kim SJ Lee SM Lee YK Oh YJ Kim YC Markelonis GJ Oh TH Systemic administration of 17beta-estradiol reduces apoptotic cell death and improves functional recovery following traumatic spinal cord injury in rats J Neurotrauma 2004 21 3 293 306 10.1089/089771504322972086 15115604

[B176] Rau SW Dubal DB Bottner M Gerhold LM Wise PM Estradiol attenuates programmed cell death after stroke-like injury J Neurosci 2003 23 36 11420 11426 14673006 10.1523/JNEUROSCI.23-36-11420.2003 PMC6740516

[B177] Sierra A Azcoitia I Garcia-Segura L Endogenous estrogen formation is neuroprotective in model of cerebellar ataxia Endocrine 2003 21 1 43 51 10.1385/ENDO:21:1:43 12777702

[B178] Matsuda J Vanier MT Saito Y Suzuki K Dramatic phenotypic improvement during pregnancy in a genetic leukodystrophy: estrogen appears to be a critical factor Hum Mol Genet 2001 10 23 2709 2715 10.1093/hmg/10.23.2709 11726558

[B179] Leranth C Roth RH Elswoth JD Naftolin F Horvath TL Redmond DE Jr Estrogen is essential for maintaining nigrostriatal dopamine neurons in primates: implications for Parkinson's disease and memory J Neurosci 2000 20 23 8604 8609 11102464 10.1523/JNEUROSCI.20-23-08604.2000 PMC6773080

[B180] Jover T Tanaka H Calderone A Oguro K Bennett MV Etgen AM Zukin RS Estrogen protects against global ischemia-induced neuronal death and prevents activation of apoptotic signaling cascades in the hippocampal CA1 J Neurosci 2002 22 6 2115 2124 11896151 10.1523/JNEUROSCI.22-06-02115.2002 PMC6758282

[B181] Dubal DB Zhu H Yu J Rau SW Shughrue PJ Merchenthaler I Kindy MS Wise PM Estrogen receptor alpha, not beta, is a critical link in estradiol-mediated protection against brain injury Proc Natl Acad Sci USA 2001 98 4 1952 1957 10.1073/pnas.041483198 11172057 PMC29363

[B182] Murphy DD Cole NB Greenberger V Segal M Estradiol increases dendritic spine density by reducing GABA neurotransmission in hippocampal neurons J Neurosci 1998 18 7 2550 2559 9502814 10.1523/JNEUROSCI.18-07-02550.1998 PMC6793090

[B183] Woolley CS Effects of estrogen in the CNS Curr Opin Neurobiol 1999 9 3 349 354 10.1016/S0959-4388(99)80051-8 10395567

[B184] Yankova M Hart SA Woolley CS Estrogen increases synaptic connectivity between single presynaptic inputs and multiple postsynaptic CA1 pyramidal cells: a serial electron-microscopic study Proc Natl Acad Sci USA 2001 98 6 3525 3530 10.1073/pnas.051624598 11248111 PMC30686

[B185] Rudick CN Woolley CS Estrogen regulates functional inhibition of hippocampal CA1 pyramidal cells in the adult female rat J Neurosci 2001 21 17 6532 6543 11517242 10.1523/JNEUROSCI.21-17-06532.2001 PMC6763095

[B186] Sandstrom NJ Williams CL Memory retention is modulated by acute estradiol and progesterone replacement Behav Neurosci 2001 115 2 384 393 10.1037/0735-7044.115.2.384 11345963

[B187] Weiss DJ Gurpide E Non-genomic effects of estrogens and antiestrogens J Steroid Biochem 1988 31 4B 671 676 10.1016/0022-4731(88)90017-9 2848981

[B188] Kuiper GG Shughrue PJ Merchenthaler I Gustafsson JA The estrogen receptor beta subtype: a novel mediator of estrogen action in neuroendocrine systems Front Neuroendocrinol 1998 19 4 253 286 10.1006/frne.1998.0170 9799586

[B189] Enmark E Gustafsson JA Oestrogen receptors - an overview J Intern Med 1999 246 2 133 138 10.1046/j.1365-2796.1999.00545.x 10447781

[B190] Kuiper GG Carlsson B Grandien K Enmark E Haggblad J Nilsson S Gustafsson JA Comparison of the ligand binding specificity and transcript tissue distribution of estrogen receptors alpha and beta Endocrinology 1997 138 3 863 870 10.1210/en.138.3.863 9048584

[B191] Nilsson S Makela S Treuter E Tujague M Thomsen J Andersson G Enmark E Pettersson K Warner M Gustafsson JA Mechanisms of estrogen action Physiol Rev 2001 81 4 1535 1565 11581496 10.1152/physrev.2001.81.4.1535

[B192] Paech K Webb P Kuiper GG Nilsson S Gustafsson J Kushner PJ Scanlan TS Differential ligand activation of estrogen receptors ERalpha and ERbeta at AP1 sites Science 1997 277 5331 1508 1510 10.1126/science.277.5331.1508 9278514

[B193] Shang Y Brown M Molecular determinants for the tissue specificity of SERMs Science 2002 295 5564 2465 2468 10.1126/science.1068537 11923541

[B194] Erlandsson MC Ohlsson C Gustafsson JA Carlsten H Role of oestrogen receptors alpha and beta in immune organ development and in oestrogen-mediated effects on thymus Immunology 2001 103 1 17 25 10.1046/j.1365-2567.2001.01212.x 11380688 PMC1783216

[B195] Igarashi H Kouro T Yokota T Comp PC Kincade PW Age and stage dependency of estrogen receptor expression by lymphocyte precursors Proc Natl Acad Sci USA 2001 98 26 15131 15136 10.1073/pnas.011513098 11752459 PMC64995

[B196] Harrington WR Sheng S Barnett DH Petz LN Katzenellenbogen JA Katzenellenbogen BS Activities of estrogen receptor alpha- and beta-selective ligands at diverse estrogen responsive gene sites mediating transactivation or transrepression Molec Cell Endocrinol 2003 206 1-2 13 22 10.1016/S0303-7207(03)00255-7 12943986

[B197] Elloso MM Phiel K Henderson RA Harris HA Adelman SJ Suppression of experimental autoimmune encephalomyelitis using estrogen receptor-selective ligands J Endocrinol 2005 185 2 243 252 10.1677/joe.1.06063 15845917

[B198] Garidou L Laffont S Douin-Echinard V Coureau C Krust A Chambon P Guery JC Estrogen receptor alpha signaling in inflammatory leukocytes is dispensable for 17beta-estradiol-mediated inhibition of experimental autoimmune encephalomyelitis J Immunol 2004 173 4 2435 2442 15294957 10.4049/jimmunol.173.4.2435

[B199] Suzuki S Brown CM Dela Cruz CD Yang E Bridwell DA Wise PM Timing of estrogen therapy after ovariectomy dictates the efficacy of its neuroprotective and antiinflammatory actions Proc Natl Acad Sci USA 2007 104 14 6013 6018 10.1073/pnas.0610394104 17389368 PMC1851608

[B200] Du S Sandoval F Trinh P Voskuhl RR Additive effects of combination treatment with anti-inflammatory and neuroprotective agents in experimental autoimmune encephalomyelitis J Neuroimmunol 2010 219 1-2 64 74 10.1016/j.jneuroim.2009.11.018 20006910 PMC3037186

[B201] Tiwari-Woodruff S Voskuhl RR Neuroprotective and anti-inflammatory effects of estrogen receptor ligand treatment in mice J Neurol Sci 2009 286 1-2 81 85 10.1016/j.jns.2009.04.023 19442988 PMC2760614

[B202] Platania P Laureanti F Bellomo M Giuffrida R Giuffrida-Stella AM Catania MV Sortino MA Differential expression of estrogen receptors alpha and beta in the spinal cord during postnatal development: localization in glial cells Neuroendocrinology 2003 77 5 334 340 10.1159/000070899 12806179

[B203] Thorogood M Hannaford PC The influence of oral contraceptives on the risk of multiple sclerosis Br J Obstet Gynaecol 1998 105 12 1296 1299 10.1111/j.1471-0528.1998.tb10008.x 9883921

[B204] Alonso A Jick SS Olek MJ Ascherio A Jick H Hernan MA Recent use of oral contraceptives and the risk of multiple sclerosis Arch Neurol 2005 62 9 1362 1365 10.1001/archneur.62.9.1362 16157743

[B205] Hernan MA Hohol MJ Olek MJ Spiegelman D Ascherio A Oral contraceptives and the incidence of multiple sclerosis Neurology 2000 55 6 848 854 10994007 10.1212/wnl.55.6.848

[B206] Da Silva JA Hall GM The effects of gender and sex hormones on outcome in rheumatoid arthritis Baillieres Clin Rheumatol 1992 6 1 196 219 1563036

[B207] Hall GM Daniels M Huskisson EC Spector TD A randomised controlled trial of the effect of hormone replacement therapy on disease activity in postmenopausal rheumatoid arthritis Ann Rheum Dis 1994 53 2 112 116 10.1136/ard.53.2.112 8129455 PMC1005262

[B208] Sicotte NL Liva SM Klutch R Pfeiffer P Bouvier S Odesa S Wu TC Voskuhl RR Treatment of multiple sclerosis with the pregnancy hormone estriol Ann Neurol 2002 52 4 421 428 10.1002/ana.10301 12325070

[B209] Soldan SS Retuerto AI Sicotte NL Voskuhl RR Immune modulation in multiple sclerosis patients treated with the pregnancy hormone estriol J Immunol 2003 171 11 6267 6274 14634144 10.4049/jimmunol.171.11.6267

[B210] Mulnard RA Cotman CW Kawas C van Dyck CH Sano M Doody R Koss E Pfeiffer E Jin S Gamst A Estrogen replacement therapy for treatment of mild to moderate Alzheimer disease: a randomized controlled trial. Alzheimer's Disease Cooperative Study [see comments] JAMA 2000 283 8 1007 1015 10.1001/jama.283.8.1007 10697060

[B211] Mulnard RA Corrada MM Kawas CH Estrogen replacement therapy, Alzheimer's disease, and mild cognitive impairment Curr Neurol Neurosci Rep 2004 4 5 368 373 10.1007/s11910-004-0083-8 15324602

[B212] Bake S Sohrabji F 17beta-estradiol differentially regulates blood-brain barrier permeability in young and aging female rats Endocrinology 2004 145 12 5471 5475 10.1210/en.2004-0984 15471968

[B213] Binder EF Schechtman KB Birge SJ Williams DB Kohrt WM Effects of hormone replacement therapy on cognitive performance in elderly women Maturitas 2001 38 2 137 146 10.1016/S0378-5122(00)00214-0 11306202

[B214] Sherwin BB Estrogen and/or androgen replacement therapy and cognitive functioning in surgically menopausal women Psychoneuroendocrinology 1988 13 4 345 357 10.1016/0306-4530(88)90060-1 3067252

[B215] Verghese J Kuslansky G Katz MJ Sliwinski M Crystal HA Buschke H Lipton RB Cognitive performance in surgically menopausal women on estrogen Neurology 2000 55 6 872 874 10994013 10.1212/wnl.55.6.872

[B216] Viscoli CM Brass LM Kernan WN Sarrel PM Suissa S Horwitz RI Estrogen therapy and risk of cognitive decline: results from the Women's Estrogen for Stroke Trial (WEST) Am J Obstet Gynecol 2005 192 2 387 393 10.1016/j.ajog.2004.08.017 15695976

[B217] Brinton RD Investigative models for determining hormone therapy-induced outcomes in brain: evidence in support of a healthy cell bias of estrogen action Ann N Y Acad Sci 2005 1052 57 74 10.1196/annals.1347.005 16024751

[B218] Manson JE Allison MA Rossouw JE Carr JJ Langer RD Hsia J Kuller LH Cochrane BB Hunt JR Ludlam SE Estrogen therapy and coronary-artery calcification NE JM 2007 356 25 2591 2602 10.1056/NEJMoa071513 17582069

[B219] Capel B Albrecht KH Washburn LL Eicher EM Migration of mesonephric cells into the mammalian gonad depends on Sry Mech Dev 1999 84 1-2 127 131 10.1016/S0925-4773(99)00047-7 10473126

[B220] Arnold AP Sex chromosomes and brain gender Nat Rev Neurosci 2004 5 9 701 708 10.1038/nrn1494 15322528

[B221] Arnold AP Burgoyne PS Are XX and XY brain cells intrinsically different? Trends Endocrinol Metab 2004 15 1 6 11 10.1016/j.tem.2003.11.001 14693420

[B222] Burgoyne PS Lovell-Badge R Rattigan A Evidence that the testis determination pathway interacts with a non-dosage compensated, X-linked gene Int J Dev Biol 2001 45 3 509 512 11417892

[B223] Carruth LL Reisert I Arnold AP Sex chromosome genes directly affect brain sexual differentiation Nat Neurosci 2002 5 10 933 934 10.1038/nn922 12244322

[B224] De Vries GJ Rissman EF Simerly RB Yang LY Scordalakes EM Auger CJ Swain A Lovell-Badge R Burgoyne PS Arnold AP A model system for study of sex chromosome effects on sexually dimorphic neural and behavioral traits J Neurosci 2002 22 20 9005 9014 12388607 10.1523/JNEUROSCI.22-20-09005.2002 PMC6757680

[B225] Markham JA Jurgens HA Auger CJ De Vries GJ Arnold AP Juraska JM Sex differences in mouse cortical thickness are independent of the complement of sex chromosomes Neuroscience 2003 116 1 71 75 10.1016/S0306-4522(02)00554-7 12535939

[B226] Wagner CK Xu J Pfau JL Quadros PS De Vries GJ Arnold AP Neonatal mice possessing an Sry transgene show a masculinized pattern of progesterone receptor expression in the brain independent of sex chromosome status Endocrinology 2004 145 3 1046 1049 10.1210/en.2003-1219 14645115

[B227] Cua DJ Hinton DR Stohlman SA Self-antigen-induced Th2 responses in experimental allergic encephalomyelitis (EAE)-resistant mice. Th2-mediated suppression of autoimmune disease J Immunol 1995 155 8 4052 4059 7561116

[B228] Cua DJ Coffman RL Stohlman SA Exposure to T helper 2 cytokines in vivo before encounter with antigen selects for T helper subsets via alterations in antigen-presenting cell function J Immunol 1996 157 7 2830 2836 8816386

[B229] Arnold AP Chen X What does the 'four core genotypes' mouse model tell us about sex differences in the brain and other tissues? Front Neuroendocrinol 2009 30 1 1 9 10.1016/j.yfrne.2008.11.001 19028515 PMC3282561

[B230] Palaszynski KM Smith DL Kamrava S Burgoyne PS Arnold AP Voskuhl RR A Yin-yang effect between sex chromosome complement and sex hormones on the immune response Endocrinology 2005 146 3280 10.1210/en.2005-0284 15905317

[B231] Smith-Bouvier DL Divekar AA Sasidhar M Du S Tiwari-Woodruff SK King JK Arnold AP Singh RR Voskuhl RR A role for sex chromosome complement in the female bias in autoimmune disease J Exp Med 2008 205 5 1099 1108 10.1084/jem.20070850 18443225 PMC2373842

[B232] Mahadevaiah SK Odorisio T Elliott DJ Rattigan A Szot M Laval SH Washburn LL McCarrey JR Cattanach BM Lovell-Badge R Mouse homologues of the human AZF candidate gene RBM are expressed in spermatogonia and spermatids, and map to a Y chromosome deletion interval associated with a high incidence of sperm abnormalities Hum Mol Genet 1998 7 4 715 727 10.1093/hmg/7.4.715 9499427

[B233] Smith DL Dong X Du S Oh M Singh RR Voskuhl RR A female preponderance for chemically induced lupus in SJL/J mice Clin Immunol 2007 122 1 101 107 10.1016/j.clim.2006.09.009 17084107 PMC2291542

[B234] Sawalha AH Harley JB Scofield RH Autoimmunity and Klinefelter's syndrome: when men have two X chromosomes J Autoimmun 2009 33 1 31 34 10.1016/j.jaut.2009.03.006 19464849 PMC2885450

[B235] Scofield RH Bruner GR Namjou B Kimberly RP Ramsey-Goldman R Petri M Reveille JD Alarcon GS Vila LM Reid J Klinefelter's syndrome (47,XXY) in male systemic lupus erythematosus patients: support for the notion of a gene-dose effect from the X chromosome Arthritis Rheum 2008 58 8 2511 2517 10.1002/art.23701 18668569 PMC2824898

